# S100A9 deletion in microglia/macrophages ameliorates brain injury through the STAT6/PPARγ pathway in ischemic stroke

**DOI:** 10.1111/cns.14881

**Published:** 2024-08-06

**Authors:** Xi Liu, Junmin Wang, Jian Jin, Qiongqiong Hu, Ting Zhao, Jian Wang, Jianbo Gao, Jiang Man

**Affiliations:** ^1^ Department of Neurology The First Affiliated Hospital of Zhengzhou University Zhengzhou China; ^2^ Department of Human Anatomy, School of Basic Medical Sciences Zhengzhou University Zhengzhou China; ^3^ MRI imaging core, Medical Research Center Third Affiliated Hospital of Zhengzhou University Zhengzhou China; ^4^ Department of Neurology, Zhengzhou Central Hospital Zhengzhou University Zhengzhou China; ^5^ Department of Neurology People's Hospital of Zhengzhou University Zhengzhou China; ^6^ Department of Radiology The First Affiliated Hospital of Zhengzhou University Zhengzhou China

**Keywords:** ischemic stroke, macrophage, microglia, neuroinflammation, phagocytosis, S100A9

## Abstract

**Background:**

Microglia and infiltrated macrophages (M/M) are integral components of the innate immune system that play a critical role in facilitating brain repair after ischemic stroke (IS) by clearing cell debris. Novel therapeutic strategies for IS therapy involve modulating M/M phenotype shifting. This study aims to elucidate the pivotal role of S100A9 in M/M and its downstream STAT6/PPARγ signaling pathway in neuroinflammation and phagocytosis after IS.

**Methods:**

In the clinical study, we initially detected the expression pattern of S100A9 in monocytes from patients with acute IS and investigated its association with the long‐term prognosis. In the in vivo study, we generated the S100A9 conditional knockout (CKO) mice and compared the stroke outcomes with the control group. We further tested the S100A9‐specific inhibitor paqunimod (PQD), for its pharmaceutical effects on stroke outcomes. Transcriptomics and in vitro studies were adopted to explore the mechanism of S100A9 in modulating the M/M phenotype, which involves the regulation of the STAT6/PPARγ signaling pathway.

**Results:**

S100A9 was predominantly expressed in classical monocytes and was correlated with unfavorable outcomes in patients of IS. S100A9 CKO mitigated infarction volume and white matter injury, enhanced cerebral blood flow and functional recovery, and prompted anti‐inflammation phenotype and efferocytosis after tMCAO. The STAT6/PPARγ pathway, an essential signaling cascade involved in immune response and inflammation, might be the downstream target mediated by S100A9 deletion, as evidenced by the STAT6 phosphorylation inhibitor AS1517499 abolishing the beneficial effect of S100A9 inhibition in tMCAO mice and cell lines. Moreover, S100A9 inhibition by PQD treatment protected against neuronal death in vitro and brain injuries in vivo.

**Conclusion:**

This study provides evidence for the first time that S100A9 in classical monocytes could potentially be a biomarker for predicting IS prognosis and reveals a novel therapeutic strategy for IS. By demonstrating that S100A9‐mediated M/M polarization and phagocytosis can be reversed by S100A9 inhibition in a STAT6/PPARγ pathway‐dependent manner, this study opens up new avenues for drug development in the field.

## Graphical abstract

The polarization imbalance between the M1/M2‐like phenotype exacerbates inflammatory response and neuronal death. By inhibiting or blocking the binding of S100A9 to TLR4, neuronal death can be reduced through modulation of inflammatory molecules. STAT6 and PPARγ, the downstream targets of TLR4, can be regulated by inhibiting or blocking S100A9.

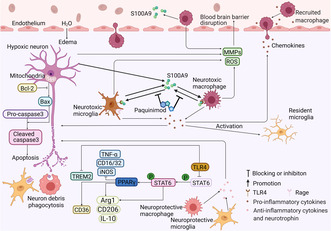

## 1 INTRODUCTION

Immediately after the ischemic stroke (IS) onset, insufficient blood flow can trigger harmful events known as the ischemic cascade, ultimately leading to irreversible cell damage. These events include excitatory toxicity, calcium overload, free radical damage, necrosis/apoptosis, and disruption of the blood–brain barrier (BBB) integrity.^1^, ^2^ Proinflammatory cells release harmful factors and cytokines that accelerate inflammatory response, contributing to progressive brain damage and neurological deficits after stroke.^3^ It is crucial to appropriately terminate these episodes to prevent further damage and promote a conducive microenvironment for brain repair.^4^ Timely removal of dead or dying cells can minimize adverse inflammation and restore brain homeostasis,^5^ thus preventing additional neuronal loss.

Microglia are a type of phagocyte naturally present in the central nervous system (CNS) and play a crucial role in regulating waste clearance and inflammatory response.^6^ Following an IS, monocytes migrate from the peripheral circulation into the brain parenchyma, differentiate into macrophages, and strengthen the immune response.^7^ Both microglia and macrophages (M/M) are highly plastic and can change their phenotype in response to brain injury.^8^ Activated M/M can be classified into proinflammatory (M1‐like) or anti‐inflammatory (M2‐like) phenotypes based on the surface markers they express.^9^, ^10^ However, the actual characteristics of M/M are difficult to define due to their complex and temporary role during the pathological process.^11^

In the early stage after stroke, newly recruited M/M mainly express more M2‐like signatures but transiently shift to the M1‐like phenotype within 1 week and persist for several weeks.^12^, ^13^ Once M1‐like phagocytes dominate the landscape, the secretion of proinflammatory factors and neurotoxic mediators increases, and effective efferocytosis is impaired.^14^ Detrimental phagocytes may remove neurons that still have the potential to recover from inflammatory injuries and survive, thereby exacerbating neuronal loss and inflicting brain atrophy.^15^, ^16^, ^17^ Therefore, regulating the balance between beneficial and detrimental profiles in the acute phase is necessary for ischemic stroke recovery.^18^ However, the potential molecular mechanisms that regulate the phagocytic function of M/M and their phenotype change after IS are currently elusive. Further research is needed to elucidate these mechanisms and develop effective therapeutic strategies for IS.

Toll‐like receptors (TLRs) are essential to the immune system, initiating an immediate response and regulating M/M polarization.^19^ In animal models, inhibiting or deleting the TLR4 gene has been found to result in smaller lesions and a reduced inflammatory response after an ischemic insult.^20^ This deletion also promotes a shift from the M1‐ to M2‐like phenotype in microglial cells.^21^ Research has shown that S100A9 (myeloid‐related protein‐14, MRP‐14), an endogenous TLR4 ligand, is vital in regulating myeloid cell function in various diseases.^22^, ^23^ Plasma S100A9 level has been closely linked to the risk, severity, mortality, and prognosis of patients with IS.^24^, ^25^ In addition, targeting S100A9 by therapeutic vaccine inhibits thrombosis without increasing the risk of bleeding in ischemic stroke mice.^26^ Therefore, investigating how S100A9 regulates the M/M phenotype in IS may provide a promising approach for stroke therapy.

In this study, we discovered that the S100A9+ classical monocytes ratio significantly increased after onset and negatively correlated with long‐term prognosis in acute IS patients. The newly generated S100A9 M/M conditional knockout (CKO) mouse and myeloid cell line were utilized to further explore the function of S100A9 in the M/M. Our in vitro and in vivo experiments demonstrated that selective deletion of S100A9 promoted M/M shifting to the anti‐inflammatory phenotype and accelerated efferocytosis in a STAT6/PPARγ pathway‐dependent manner. Additionally, we found that paquinimod (PQD), a selective binding blocker of S100A9/TRL4, mitigated brain damage and improved the outcomes in wildtype tMCAO mice. Blocking S100A9 by PQD could be an alternative intervention approach besides blood vessel recanalization.

## 2 MATERIALS AND METHODS

### 2.1 Clinical subjects

Thirty‐one patients diagnosed with acute ischemic stroke (AIS) of the anterior circulation within 24 h of symptom onset and age were recruited as the AIS group. The blood was taken within 1 day after inpatient adoption. Sex‐matched healthy individuals (*n* = 25) without any neurological disease history were recruited as the control group. The National Institute of Health Stroke Scale (NIHSS) score at adoption and modified Rankin Scale (mRS) score at 90 days after stroke onset were recorded. The inclusion and exclusion criteria set followed previous publications.^27^

### 2.2 Flow cytometry analysis and cell sorting for transcriptomics analysis

Peripheral blood monocytes (PBMCs) from AIS patients or healthy controls were isolated by centrifugation using Ficoll‐Paque solution (Cytiva). The cells were stained with anti‐CD16‐APC and anti‐CD14‐FITC antibodies or isotype controls at 4°C for 30 min, followed by permeabilization and stained with anti‐S100A9‐PE for final detection.

For M/M extraction, single‐cell suspensions of sham or tMCAO S100A9 CKO mouse brain were stained with fluorescent‐conjugated antibodies and subjected to flow‐cytometry sorting with an Aria FACS cytometer (BD Biosciences). The harvested M/M was lysed in TRIzol reagent (Solarbio) for RNA extraction and transcriptomic analysis (Lianchuan Bioscience).

### 2.3 Animal experiments

We followed the ARRIVE guidelines^28^ to conduct and report the animal study. Every effort was made to minimize the number of mice used and prevent potential suffering. All mice used in the study were male to avoid any potential confounding variation caused by sex differences and female estrous cycle and to prevent the protective effects of estrogen in an ischemic stroke study. The mice were housed in an SPF‐grade facility with a 12 h light/dark cycle and could access food and water.

#### 2.3.1 Generation of S100A9 conditional knockout mice

To generate M/M‐specific S100A9‐deficient mice, the S100A9^fl^/^fl^ mice (stock no: S‐CKO‐04902, Caygen), which carry loxP sites flanking target exons of the S100A9 gene were crossed with mice that express Cre recombinase by the Cx3cr1 (chemokine (C‐X3‐C motif) receptor 1) promoter (stock no: C001032, Caygen) (Figure S1A). The offspring including S100A9^fl^/^fl^ Cx3cr1Cre^+^/^−^ (S100A9 CKO) and littermate S100A9^fl^/^fl^ Cx3cr1Cre^−^/^−^ (control). All mice used in the experiment were confirmed by genotyping (Figure S1).

#### 2.3.2 Sample size estimation and experimental grouping

We followed the STAIR guidelines^29^ in the study design. Sample sizes (*n* = 6 mice per group) were estimated according to our pilot study, and all animals were randomly allocated to different groups by the randomization software (https://www.randomizer.org/).^30^ Investigators blinded to the grouping conducted data analysis.

#### 2.3.3 Transient middle cerebral artery occlusion (tMCAO) and drug treatment

The tMCAO was carried out according to previous publications.^31^ Briefly, anesthesia was performed by inhalation of 3% isoflurane (RWD, Shenzhen) and maintained at 1.5% during the surgical procedure. After an incision was made on the right common carotid artery, the monofilament (Cinontech Corp) was advanced via the internal carotid artery until it blocked the middle cerebral artery. After 40 min of occlusion, the monofilament was slowly withdrawn for recanalization. The sham group underwent the same surgery except for the insertion procedure. For drug treatment, the BBB penetrable S100A9 specific inhibitor paquinimod was administered intraperitoneally at a dosage of 8 mg/kg/day for 7 consecutive days,^32^ and the STAT6 phosphoration‐inhibitor AS1517499 (Sigma–Aldrich) was administered intranasally at a dose of 2.6 mg/kg/day for 3 consecutive days, according to a previous publication.^33^ The control group received an equal volume of vehicle treatment. After the operation, the animals were returned to their home cages and carefully monitored during the study.

#### 2.3.4 Neurologic function assessment and behavioral test

An investigator blinded to the treatment group performed the neurologic function assessment. The modified neurological severity score (NSS), including motor, sensory, and reflex tests, was performed as described^34^ on 3, 7, 14, and 21 days after tMCAO. Each test was performed in triplicate to obtain a mean score.

To guarantee accurate results, all mice underwent pre‐operative walk training to acclimate to the Catwalk (Noldus, CatWalk XT 9.1) system. The catwalk gait analysis tests were performed on day 21 after tMCAO as previously described protocols,^35^ a battery of gait parameters including run characterization, locomotion, and temporal parameters were recorded and analyzed. The Morris water maze (MWM) test assessed cognitive function recovery on day 28 after tMCAO as per previous publication.^36^ Movement track (path length), time taken to reach the platform area (escape latency), and percentage of time spent in the goal quadrant were processed by the Any‐Maze software (Stoelting Co).

#### 2.3.5 Laser speckle contrast imaging (LSCI)

CBF was measured using the LSCI System (RWD) at different time intervals after tMCAO. To evaluate blood flow, regions of interest (ROIs) were selected symmetrically on the bilateral brain hemisphere where the laser was projected. The ratio of flux values between the contralateral and ipsilateral ROIs was calculated.^37^

#### 2.3.6 High‐field MRI detection for lesion volume, brain swelling, and BBB integrity assessment

Multi‐modal MRI was performed using a 4.7‐T MRI scanner (MR solutions) equipped with a mouse head coil on days 3, 7, and 14 after tMCAO as previously described methodology.^38^ The T2‐weighted images (T2WI) were captured with a thickness of 0.7 mm using a spin echo sequence (repetition time (TR)/echo time (TE) = 5000/50 ms, 16 slices with voxel size = 256 × 252 × 700 μm FOV = 22 mm). Corrected brain infarct volume (CIV) was calculated as the previous description^39^ by using the following equation: [Vch‐(Vih‐Vinf)/Vch × 100%, Vch = contralateral hemisphere volume, Vih = ipsilateral hemisphere volume, Vinf = infarction volume]. Corrected brain edema (CBE) was calculated using the following equation: (Vih‐Vch)/Vch × 100%. After T1WI acquisition, the GD‐DTPA (1 mmol/kg, Bayer AG) was intraperitoneally injected in the bolus. Post‐contrast agent (CA) T1WI was acquired with the same parameters to assess mouse brain BBB integrity. The ΔΤ1‐map was processed by subtracting the image of pre‐Gd T1WI from post‐Gd T1WI by ImageJ Fiji software (NIH) equipped with MRI plugins.^40^

### 2.4 Tissue and cell immunofluorescence microscopy

Following our established protocol,^41^ the mouse's brain sections were stained with primary antibodies and corresponding fluorescent secondary antibodies. Infarct volume was determined by Nissl staining (Beyotime). Phagocytosis of dead/dying neurons was determined by NeuN/Iba1 double‐labeled or NeuN/Iba1/TUNEL triple‐labeled cells in the peri‐infarct area. Following confluence and treatment, the cultured cells were subjected to immunostaining. Cell death in brain tissue and cultured cells was detected using a TUNEL staining kit (Beyotime). Primary antibodies and the dilutions used in the experiment are listed in (Table S1). Images were captured using a Leica confocal microscope (Leica TCS SP8) and analyzed by a technician blinded to the grouping.

### 2.5 Western blot analysis

By our established protocol,^34^ an equal amount of protein was loaded onto SDS–PAGE gels for electrophoresis and transferred onto PVDF membranes (Millipore). After milk blockage and incubation with the designated antibody (Table S1), the target protein was detected with corresponding horseradish peroxidase (HRP)‐conjugated secondary antibodies. Protein bands were visualized with enhanced chemiluminescence (ECL) reagents and captured with an imaging system (ClinX Science Instruments). Band intensity was normalized with the internal control and quantified by ImageJ software (NIH). Independent experiments were repeated in triplicate.

### 2.6 Cell culture and drug treatment

The mouse microglial stable cell line, BV2, and neuronal cell line HT22 were cultured in high‐glucose DMEM (Invitrogen) supplemented with 10% fetal bovine serum. The cells were incubated in a humidified incubator with 5% CO2 at 37°C. To establish oxygen and glucose deprivation/reoxygenation (OGD/R), the cells were cultured in a glucose‐free medium in a low oxygen chamber (1% O_2_, 94% N_2_, and 5% CO_2_) at 37°C for 6 h, followed by normal oxygen conditions for 12 h before harvesting. For phagocytosis assay, OGD/R‐treated HT22 cells were incubated with 1 μg/mL propidium iodide (PI, Solarbio, China) in an FBS‐free medium at 37°C for 30 min to label the dead cells. After washing 3 times, the cells were added to BV2 culture dishes at a quantitative ratio of 5:1 and incubated for another 6 h. The dextran phagocytosis assay was performed by treating BV2 cells with FITC‐dextran (MW ~ 40,000, Abmole) at a final concentration of 0.5 mg/mL for 30 min after OGD/R and washed 3 times before harvesting.

For drug treatment, the BV2 cells were seeded into the upper chamber of co‐culture plates (0.4 μm pore size, Corning) with OGD/R HT22 cells in the lower chamber. HT22 cells co‐cultured with BV2 underwent normoxic conditions and served as controls. Paquinimod (Sigma–Aldrich) or AS1517499 (100 nM) were added into the medium 12 h after OGD/R before harvesting.

### 2.7 S100a9 siRNA gene knockdown and semiquantitative RT–PCR

A pool of 3 sequence‐specific 19–25 nt siRNAs (sc‐43,345, Santa‐Cruz Biotechnology) targeting mouse S100A9 was transfected into the BV2 cells. The scrambled siRNA (sc‐37,007) was used as a negative control. Cells were subjected to downstream experiments 48 h after transfection. A one‐step RT‐PCR system (Invitrogen) was used to reverse transcription and DNA amplification. A 114‐bp fragment of the mouse *S100A9* gene was amplified, and a 249‐bp fragment of the mouse GAPDH gene served as an internal control (Table S2). The PCR products were resolved on 1.5% agarose gels and analyzed by the Gel EZ Imaging system (Bio‐Rad).

### 2.8 Statistical analysis

GraphPad Prism 9 software was used for statistical analysis. All data are expressed as means ± SDs. Parameter tests were performed by unpaired Student's *t‐test* for two groups or one‐way analysis of variance (ANOVA) or two‐way ANOVA followed by Tukey's or Holm‐Sídak's multiple comparison test (for normally distributed data) and the *U* test or Kruskal–Wallis test followed by Dunn's multiple comparison test (for nonnormally distributed data). *p* < 0.05 was considered statistically significant.

## 3 RESULTS

### 3.1 The S100A9 ^+^ classical monocyte population increased following stroke and was associated with long‐term unfavorable outcomes in patients with AIS

**FIGURE 1:**
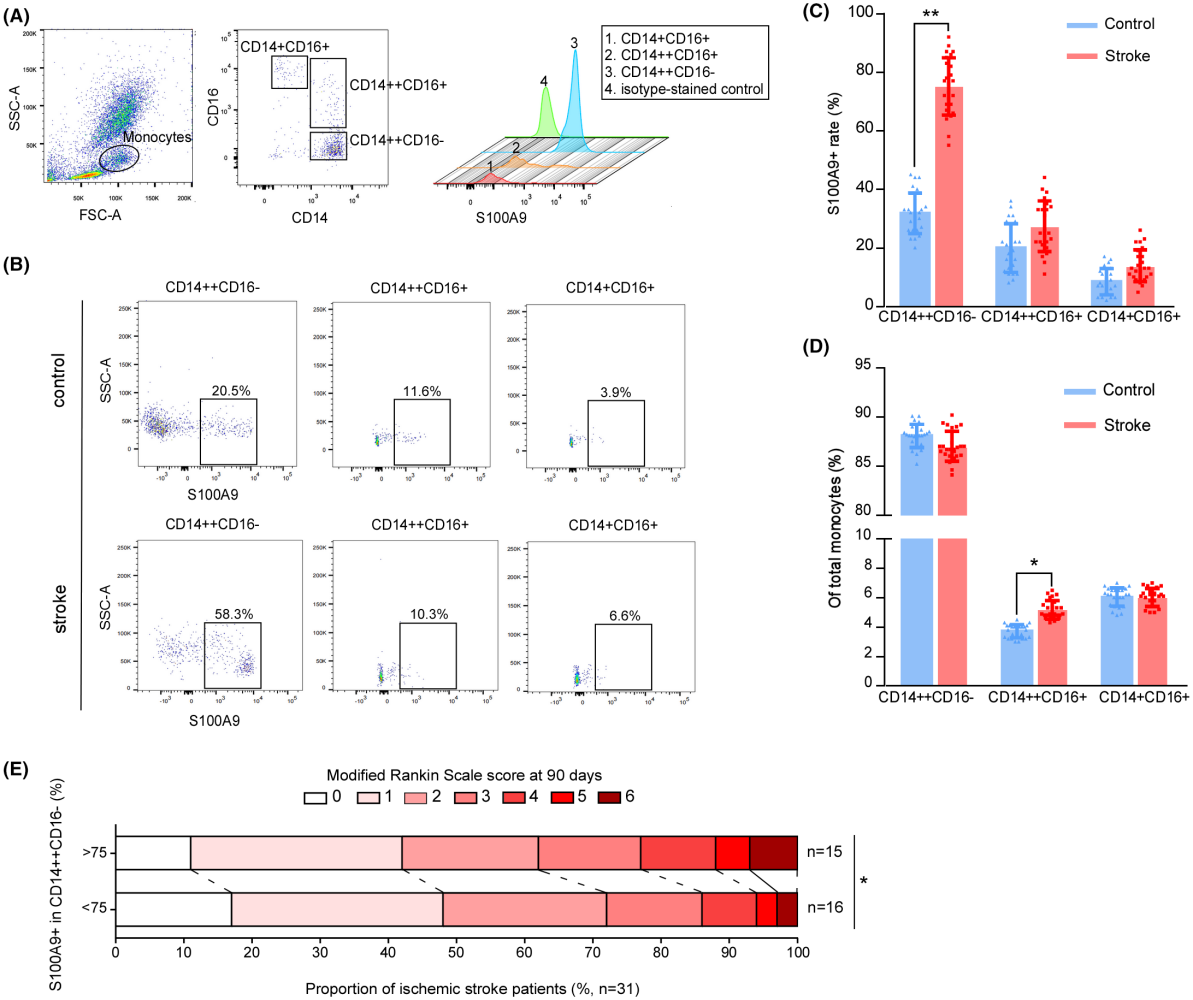
Intracellular S100A9 expression pattern in monocyte subpopulation and its correlation with clinical prognosis. (A) Flow cytometry images show the gating stratagem of monocyte subpopulations based on the expression of CD16 and CD14. The CD14^++^CD16^−^classical monocyte was the primary source of intracellular S100A9. (B) Intracellular expression pattern of S100A9 in each monocyte subpopulation under healthy (upper panel) and IS (lower panel) conditions. (C) Quantification of S100A9‐positive cells in each subpopulation under healthy and IS conditions. The percentage of S100A9‐positive cells increased significantly in patients' CD14^++^CD16^−^ classical monocyte subpopulation after a stroke. In contrast, the other monocyte subpopulations exhibited no significant change in S100A9 expression levels. **p < 0.01. (D) Quantifying the ratio of each subpopulation to total monocytes in healthy controls and IS patients. The CD14^++^CD16^+^ intermediate monocyte population increased after the stroke. *p < 0.05. (E) The modified Rankin Scale was used to determine the clinical progress of the patients at 90 days, with scores ranging from 0 to 6, with 0 indicating no symptoms, 1 indicating no clinically significant disability, 2 indicating slight disability, 3 indicating moderate disability, 4 indicating moderately severe disability, 5 indicating severe disability, and 6 indicating deaths. The numbers indicate the proportion of patients (%) per category.

Elevated counts of peripheral monocytes have been linked with worse disease severity and prognosis in patients diagnosed with acute ischemic stroke (AIS).^42^ Here, we observed that the CD14^++^CD16^−^ classical sub‐population expresses more S100A9 among the monocyte populations under healthy conditions (Figure 1A). Notably, the S100A9 expression rate increased significantly in classical monocytes but not in the intermediate CD14^++^/CD16^+^ and the nonclassical CD14^+^/CD16^+^ subsets in AIS patients (Figure 1B,C). To preclude the possibility of total monocyte count variation caused by stroke, we quantified the sub‐population to the total monocyte ratio. Our data, which is in line with a recent study,^43^ show that the percentage of intermediate monocyte subsets increased. In contrast, the rates of the classical and nonclassical populations remained stable after AIS (Figure 1D).

In the post‐stroke follow‐up, we found that the ratio of S100A9^+^ cells in classical monocytes in the acute phase was positively correlated with the modified ranking scale score at 90 days after stroke onset (Figure 1E). This result indicated that a higher level of S100A9 in classical monocytes may have an adverse outcome in AIS patients. Our clinical investigations, taken together, provided evidence that the ratio of S100A9^+^ to total classical monocytes could serve as a potential biomarker for prognosis prediction.

### 3.2 S100A9 CKO mice exhibited improved cerebral blood flow, reduced infarct area, and improved neurofunction after tMCAO

**FIGURE 2:**
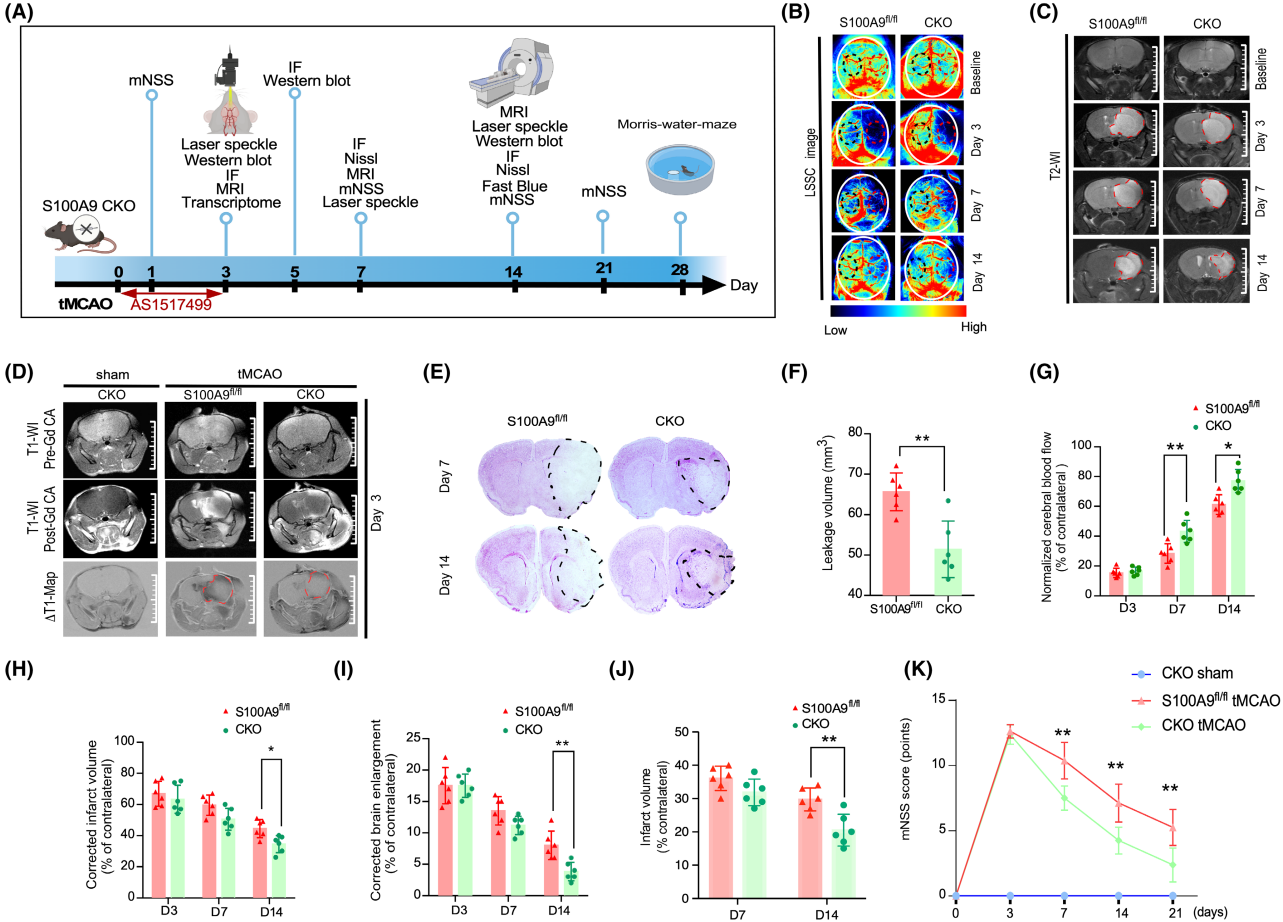
S100A9 CKO improves cerebral blood flow, reduces infarct area, and improves neurologic outcome after tMCAO. (A) The schematic diagram illustrates the experimental procedure. (B) The cerebral blood flow after tMCAO was assessed at different time points in S100A9 CKO and control mice using Lasser sparkle contrast imaging, which was semi‐quantified by normalizing with contralateral blood flow (G). (C) The lesion area was evaluated by T2‐WI magnetic resonance imaging (MRI) at baseline and on days 3, 7, and 14 after tMCAO. Corrected infarct volume (H) and brain enlargement (I) were normalized to the contralateral hemisphere. (D) The injection of the Gd contrast agent was shown in a representative T1‐WI MRI before (top panel) and after (bottom panel) on day 3 after tMCAO. ΔΤ1‐map (lower panel) processed with ImageJ software was used to determine the BBB leakage area on each slide. S100A9 CKO mice exhibited significantly reduced leakage volume compared to the control group, **p < 0.01 (F). (E) Brain sections were stained with Luxol Fast blue to evaluate infarct volume in S100A9 CKO and control mice on days 7 and 14 after tMCAO. The infarct tissue unstained with Luxol fast blue is outlined by a black dashed line. (J) Infarct volume was quantified by normalizing to the contralateral hemisphere. *p < 0.05. (K) The neurologic deficits of S100A9 CKO and control mice were assessed at different time points after tMCAO using the mNSS scoring. *p < 0.05.

The expression of S100A9 and its receptor TLR4 was punctually localized in the Iba‐1^+^ cells but not the NeuN^+^ or GFAP^+^ cells on day 3 after tMCAO (Figure S1C). Consistent with a previous report,^43^ our findings confirmed that S100A9 is mainly expressed by microglia and infiltrating macrophages in the brain. To further explore the function of S100A9 in the M/M cells, we generated S100A9 conditional knocked‐out (CKO) mice by crossing S100A9^fl^/^fl^ mice with CX3CR1 Cre^+^/^+^ transgenic mice. All mice were genotyped to determine the *CX3CR1 Cre* and *S100A9 flox* expression (Figure S1B). Specific deletion of S100A9 was further confirmed by immunostaining of S100A9 in the brain (Figure S1D). Compared with the control group, the S100A9 CKO tMCAO group displayed significant improvement in cerebral blood flow on day 7 by LSC imaging (Figure 2B,G), reduced BBB leakage on day 3 by ΔT1 map (Figure 2D,F), and reduced corrected infarct volume(Figure 2C,H) and brain swelling (Figure 2C,I) on day 14 by T2WI. The lesion volume was confirmed by Nissl staining (Figure 2E,J). The mNSS evaluation indicated that S100A9 CKO mice displayed improved neurofunction (Figure 2K). In contrast, there was no significant difference in lesion volume or neurologic deficits between CX3CR1Cre^+^/^+^ and wildtype mice, which ruled out the nonspecific effects of cre recombinase on stroke outcome (data not shown).

### 3.3 S100A9 CKO facilitated M/M phenotype shifting toward an anti‐inflammatory profile and promoted M/M engulfment of apoptotic cells after tMCAO

**FIGURE 3:**
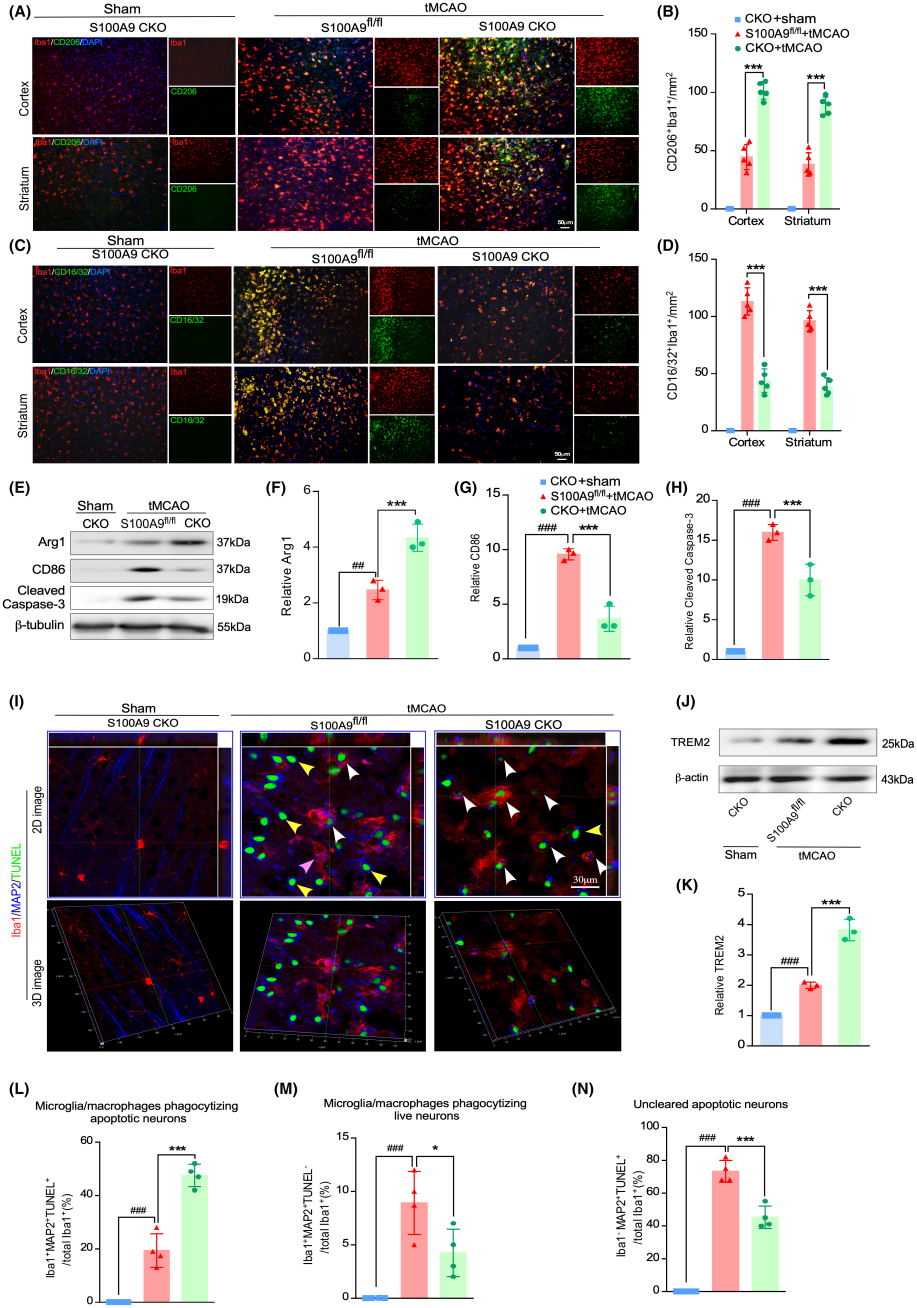
S100A9 CKO promotes M/M anti‐inflammatory polarization and augments efferocytosis after tMCAO. (A) The peri‐infarct area, comprising the cortex (upper panel) and striatum (lower panel) underwent co‐immunostaining with CD206 and Iba‐1. (B) The CD206^+^/Iba‐1^+^ ratio was quantified in the cortex and striatum of the S100A9 CKO and control groups. The CD206^+^/Iba‐1^+^ ratio increased significantly in the penumbra of S100A9 CKO mice. ***p < 0.01 (A) (C) Co‐immunostaining of CD16/32 and Iba‐1 in the cortex (upper panel) and the striatum (lower panel) in the peri‐infarct area. (B) Quantification of the CD16/32^+^/Iba‐1^+^ ratio in the cortex and striatum of the S100A9 CKO and control groups. The CD16/32^+^/Iba‐1^+^ ratio decreased significantly in the penumbra of S100A9 CKO mice. ***p < 0.01. (E) Immunoblots were conducted to detect Arg‐1, CD86, and cleaved caspase‐3 protein levels in brain tissue from the peri‐infarct area of each group. Protein expression levels were normalized to the internal control β‐tubulin. Quantification of protein expression of Arg‐1 (F), CD86 (G), and cleaved caspase‐3 (H). ^###^ *p* < 0.001vs. sham, and ***p < 0.001vs. S100A9 CKO. (I) Immunofluorescence staining of MAP2, Iba‐1, and TUNEL in the peri‐infarct area of the different groups (2D image, upper panel). The successful efferocytosis (dying neurons taken up by M/M, indicated by white arrows) and mistaken efferocytosis (live neuron taken up by the M/M, indicated by yellow arrow) were confirmed by the 3D stack image (lower panel). Quantification of the successful efferocytosis ratio (L), mistaken phagocytosis ratio (M), and uncleared dying neuron ratio (N) ^###^ *p* < 0.001. sham, *p < 0.05, ***p < 0.001vs. S100A9 CKO. (J) The phagocytosis marker TREM2 in the peri‐infarct region was detected by Western blotting, and protein expression levels were normalized to the internal control β‐tubulin. (K) Quantification of the relative expression of TREM2 in each group. ^##^ *p* < 0.001. sham, **p < 0.01vs. S100A9 CKO.

The S100A9 CKO tMCAO mice manifested a significant increase in the percentage of cells that were double‐positive for the M2‐like marker CD206 (Figure 3A,B) and a decrease for the M1‐like marker CD16/32 co‐stained with Iba‐1 in both the cortex and the striatum compared to the control (Figure 3C,D) on day3 after tMCAO. Additionally, Western blot revealed that the S100A9 CKO group significantly increased the M2‐like marker Arg1 and decreased the M1‐like marker CD86 and caspase‐3 compared with the control (Figure 3E,F–H). These data indicate that S100A9 CKO affects the M/M phenotype shifting after tMCAO.

Removing dead/dying cells is crucial in resolving inflammation and restoring brain homeostasis following stroke. To investigate if S100A9 plays a critical role in efferocytosis in IS, a comparative analysis of efferocytosis was conducted on day 5 after tMCAO. Effective efferocytosis was determined by double‐positive for TUNEL (cell death marker) and MAP2 (neuronal marker) within Iba1^+^ cells in the lesion area according to a previous publication.^44^ No TNUEL^+^MAP2^+^ cells were observed in the sham mouse brain (Figure 3I, left panel), indicating that S100A9 CKO does not affect phagocytosis under physiological conditions. However, triple‐positive TNUEL/MAP2/Iba‐1 cells increased significantly in the CKO group compared with the control (Figure 3L). These results suggested that S100A9 CKO promotes efferocytosis (Figures 3I right panel). Conversely, the numbers of Iba‐1^+^/MAP2^+^/TUNEL^−^ cells (Figure 3M) and Iba‐1^−^MAP2^+^/TUNEL^+^ cells (Figure 3N) were higher in the control group, indicating increased mistaken efferocytosis and accumulation of uncleared dead/dying neurons. The phagocytosis marker TREM2 significantly increased in the ischemic brain of the CKO compared with the control (Figure 3J,K). These findings suggest that deleting S100A9 reduced neuronal cell death, facilitated the shift of M/M polarization to an anti‐inflammatory phenotype, and promoted efferocytosis after tMCAO.

### 3.4 S100A9 CKO mitigated white matter injury and cognitive impairment after tMCAO

**FIGURE 4:**
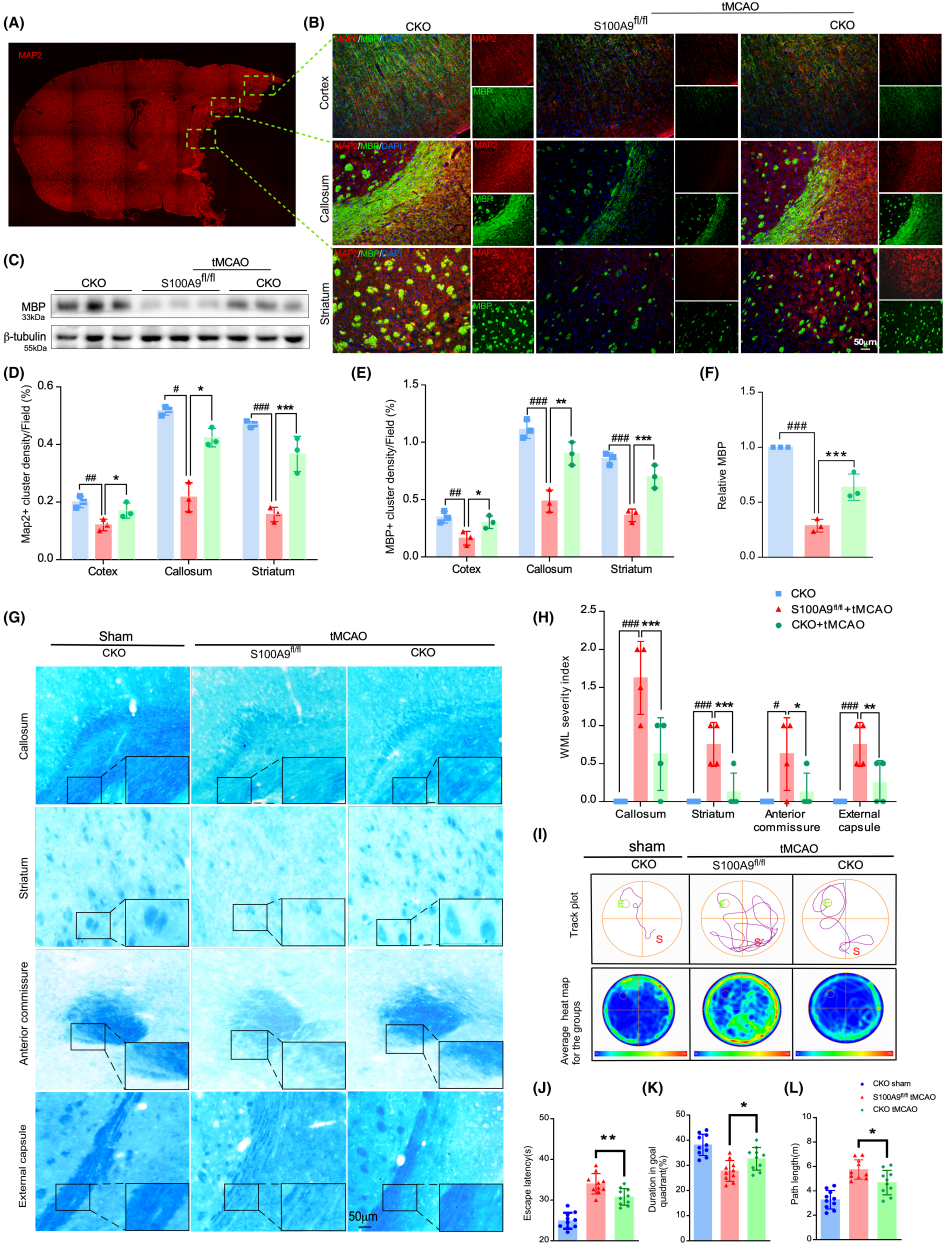
S100A9 CKO protects against white matter lesions and cognitive impairment after tMCAO. (A) A brain slice scan to indicate ROI selection. (B) Co‐immunostaining of MAP2 and MBP in the cortex (right upper panel), callosum (right middle panel), and striatum (right lower panel) in the peri‐infarct area. The immunoactivity of the MAP2 (D) and MBP (E) clusters was normalized in the contralateral hemisphere and quantified in each group. (C) MBP expression in the infarcted hemisphere was detected by Western blotting. Protein expression in each group was quantified and normalized to that of the internal control β‐tubulin (F). (G) Fast blue staining of the axon density in the callosum, striatum, anterior commissure, and external capsule in each group. (H) The quantification of axon density was normalized to the contralateral hemisphere and expressed as the WML severity index. *p < 0.05, ^#^ *p* < 0.05, **p < 0.01, ^###^ *p* < 0.001, ***p < 0.001. (I) Representative track plots (upper panel) and group heatmap (lower panel) of mice in each group in the Morris water maze test were analyzed. Escape latency (J), duration of the goal quadrant (K), and length of the swimming path (L) were quantified. *p < 0.05.

M/M is essential in developing white matter (WM) injury and subsequent repair after stroke.^45^ To further explore the implications of S100A9 CKO on WM integrity after IS, the myelin marker MBP and the neurofilament marker MAP2 expression in the different brain areas were co‐stained on day 21 after tMCAO (Figure 4A). The MAP2 and MBP immunoactivity in the control group decreased significantly compared with the sham in the different areas (Figure 4B), but increased in the S100A9 CKO group (Figure 4D,E). MBP protein levels in the lesioned hemisphere detected by Western blotting also suggested preserved WM integrity by S100A9 CKO after tMCAO (Figure 4C,F). Similarly, the myelin density in different areas determined by Luxol‐fast‐blue (LFB) staining (Figure 4G) was significantly reduced in the S100A9 CKO group compared to the S100A9^fl/fl^ group (Figure 4H), indicating a mitigated myelin loss by S100A9 CKO.

Given that WM integrity is essential for cognitive function, we investigated mental recovery using the MWM test (Figure 4I). The S100A9 CKO mice exhibited a shorter escape latency and path length time but spent more time in the goal quadrant than the control (Figure 4J,K,L). However, no significant difference in average cruise speed was observed among the groups, which ruled out the confounding possibility by locomotive deficits. These data suggest that S100A9 CKO effectively attenuates white matter loss and cognitive impairment after tMCAO.

### 3.5 Transcriptomic analysis showed unique transcriptomic patterns of S100A9 CKO after tMCAO

To explore the potential signaling pathways mediated by the beneficial role of S100A9 CKO in IS. M/M cells harvested from the ischemic brain hemisphere of the S100A9^fl^/^fl^ or S100A9 CKO mice were (Figure S2A) subjected to transcriptome analysis. (Figure S2B). Notably, among the screened differential expression genes, *STAT1*, which is known to modulate the microglial phenotype dependent on TLR4,^46^ was downregulated in the CKO group. Recent studies have shown that STAT1 antagonistic regulator STAT6 knockout prompted M/M toward a proinflammatory phenotype, along with impaired clearance of dead/dying neurons and increased cerebral inflammation and neuronal death in stroke mice.^44^ Presumably, the beneficial effect of S100A9 CKO after tMCAO is linked to the downregulation of genes associated with inflammation and apoptosis and the modulation of STAT1 and STAT6.

### 3.6 S100A9 CKO in M/M protects against inflammation‐associated brain injury in a STAT6/PPARγ‐dependent manner

**FIGURE 5:**
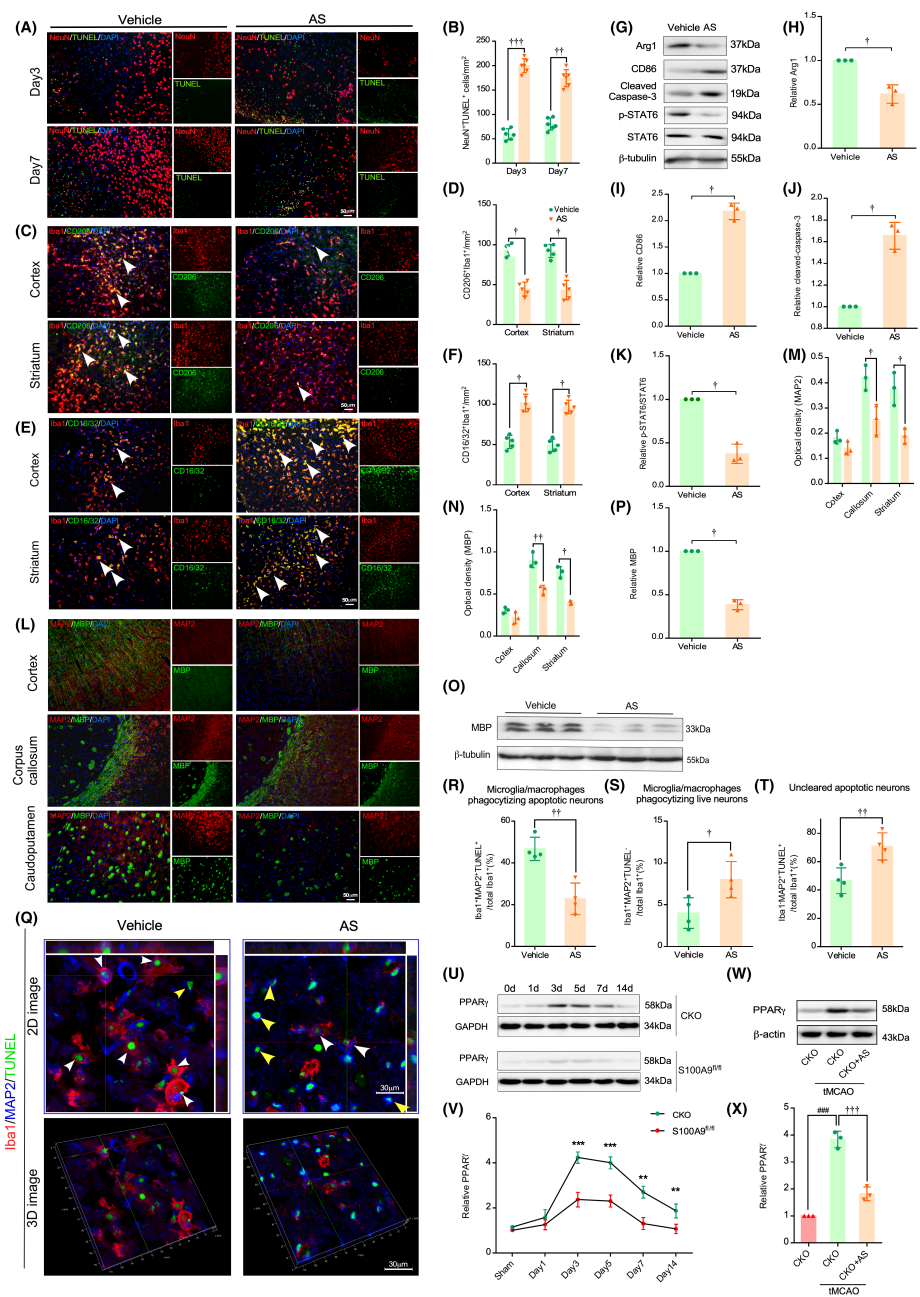
STAT6/PPARγ is involved in the protective effect of S100A9 CKO in tMCAO. (A) Immunofluorescence co‐staining of NeuN and TUNEL in the peri‐infarct area of S100A9 CKO mice treated with vehicle or AS was performed 3 and 7 days after tMCAO. (B) Quantification of NeuN^+^/TUNEL^+^ cells in the peri‐infarct area in each group. ^††^ *p* < 0.01, ^†††^ *p* < 0.01 vs. vehicle. (C) Immunofluorescence co‐staining and quantification (D) of CD206 and Iba‐1 in the cortex (upper panel) and striatum (lower panel) in vehicle‐ or AS‐treated S100A9 CKO mice on day 3 after tMCAO. (E) Immunofluorescence was co‐staining and quantification (F) of CD16/32 and Iba‐1 in the cortex and striatum in vehicle‐ or AS‐treated S100A9 CKO mice on day 3 after tMCAO. (G) Western blot detected Arg1, CD86, cleaved Caspase‐3, STAT6, and pSTAT6 in the infarcted brain hemisphere in vehicle‐ or AS‐treated CKO mice 3 days after tMCAO, protein expression levels were quantified in (H‐K). ^†^ *p* < 0.05 vs. vehicle. (L) The expression of MBP and MAP2 in different areas of the brains of S100A9 CKO mice treated with vehicle or AS on day 21 after tMCAO was quantified (M,N). ^†^ *p* < 0.05, ^†^ *p* < 0.01 vs. vehicle. MBP expression (O) in the infarcted hemisphere and was quantified in (P). ^†^ *p* < 0.05. (Q) Staining of MAP2, Iba‐1, and the cell death marker TUNEL in the peri‐infarct area of sham or tMCAO CKO mice (2D image, upper panel) and 3D stack image (lower panel). Dead neurons phagocytosed by M/M (white arrows) and live neurons phagocytosed by M/M (yellow arrows) were quantified as successful efferocytosis ratio (R), mistaken phagocytosis ratio (S), and unclear dying neuron ratio (T) ^†^ *p* < 0.05, ^††^ *p* < 0.01. (U) The expression of PPARγ in the infarcted hemisphere of CKO or control mice at 0, 1, 3, 5, 7, and 14 days after tMCAO was detected and quantified by WB and quantified in (V) **p < 0.05, ***p < 0. 001vs.S100A9^fl^/^fl^. (W) PPARγ expression in the infarcted hemisphere of sham, vehicle‐, or AS‐treated mouse brain hemispheres of CKO tMCAO CKO was detected by WB and quantified in (X). ^†††^ *p* < 0.01 vs. CKO + vehicle, ^###^ *p* < 0.01 vs. CKO sham.

To testify if STAT6 is the potential downstream target by S100A9 CKO, the S100A9 CKO mice were treated with STAT6 phosphorylation‐specific inhibitor AS1517499 (AS) or vehicle for 3 consecutive days after tMCAO. AS treatment had a detrimental effect on the protective effect of S100A9 CKO, as evidenced by a significant increase in the number of TUNEL^+^ dead/dying cells in the AS‐treated group compared to the vehicle‐treated group on days 3 and 7 after tMCAO (Figures 5A,B). Furthermore, the cell counts of CD206^+^/Iba‐1^+^ decreased, while CD16/32^+^/Iba‐1^+^ increased in both the cortex and striatum (Figures 5C,E) on day 3 after tMCAO. Protein expression levels in the infarcted brain hemisphere (Figure 5G) showed that CD32 and cleaved caspase3 increased in the AS treatment group, whereas Arg‐1 and pSTAT6 decreased (Figures 5H–K), whereas the STAT6 holoprotein remained unaffected. Similarly, AS treatment also reduced the expression of MAP2 and MBP and axon density in the ischemic brain (Figures 5L,M–P). In addition, AS treatment also impaired efferocytosis, as evidenced by decreased MAP2^+^TUNEL^+^ Iba‐1^+^ triple‐positive cells and increased MAP2^+^TUNEL^−^Iba‐1^+^ mistakenly removed cells and MAP2^+^TUNEL^+^Iba‐1^−^ incompetent phagocytosis in the AS‐treated groups compared with the vehicle group (Figures 5Q,R–T). In summary, inhibition of STAT6 phosphorylation impairs the protective effect of S100A9 CKO.

Evidence has shown that the peroxisome proliferator‐activated receptor (PPARγ), a transcriptional target downstream of STAT6,^47^ whose expression in macrophages could facilitate inflammation resolution.^40^ To exemplify if S100A9 CKO, mechanically, by targeting the STAT6/PPARγ signal pathway, we outlined the PPARγ protein expression time course in both S100A9 CKO and control groups at various time points post‐tMCAO (Figure 5U). PPARγ expression significantly increased in both groups after tMCAO peaked on day 3, and gradually decreased, finally reaching the baseline level around day 14. Interestingly, compared with the S100A9^fl^/^fl^ group, S100A9 CKO increased the expression level of PPARγ on days 3, 5, 7, and 14 (Figure 5V). The protein expression of PPARγ in S100A9 CKO mice was significantly increased after tMCAO compared to sham groups and decreased by STAT6 phosphorylation inhibition (Figure 5W). Our results support that S100A9 CKO modulates the M/M immune response in a STAT6/PPARγ‐dependent manner.

### 3.7 Downregulation of S100A9 promotes M/M cell polarization toward an anti‐inflammatory phenotype and decreases inflammation‐associated neuron death in response to OGD/R

We aim to confirm our findings in vivo and to explore the mechanism underlying the S100A9 inhibition in vitro further. The microglial cell line BV2 cells were exposed to OGD/R to mimic ischemia/reperfusion injury, followed by treatment with different doses of the S100A9 inhibitor, laquinimod (PQD). The protein assay results showed that the typical M2‐like cell marker Arg‐1 and the anti‐inflammatory cytokine Il‐10 increased. In contrast, the M1‐like markers CD86 and the pro‐inflammatory cytokine TNF‐α decreased dose‐dependently after PQD treatment compared to vehicle treatment (Figure S3A,B–E).

To avoid the off‐target effect of the drug treatment and ensure the observed effects were due to the inhibition of S100A9, the S100A9 gene was knocked down by a pool of siRNA transfection into the cell line. Successful gene silencing was confirmed by RT‐PCR and Western blot (Figure S3D,E). Silencing of the S*100A9* gene enhanced anti‐inflammatory Arg1 expression (Figure S3F,N), whereas it decreased the expression of pro‐inflammatory iNOS in response to OGD/R stress (Figure S3G,O).

Furthermore, we sought to investigate whether disruption of S100A9 could alleviate neurotoxicity of the microglial cell line. The HT22 cells were co‐cultured with BV2 cells subjected to OGD/R stress to mimic the in vivo glia–neuron interactions. Protein assay revealed that knocking down of *S100A9* in the BV2 cells rescued cell death of co‐cultured neuronal HT22 cells (Figure S3H). Compared with the control, the cleaved caspase‐3 and BAX in HT22 significantly increased after OGD/R in the vehicles or scrambled siRNA‐treated groups. However, the increasing trend of cleaved caspase‐3 and BAX was reversed by *S100A9* siRNA or PQD treatment compared with vehicle or scramble siRNA groups. The anti‐apoptotic marker Bcl‐2 was increased by treatment with S100A9 siRNA or PQD compared with vehicle or scrambled siRNA‐treated groups, respectively (Figure S3I–K). Overall, these findings suggest that downregulation or inhibition of S100A9 in M/M cells not only attenuates the pro‐inflammatory phenotype but also ameliorates inflammation‐induced neuronal death, indicating a neuroprotective effect of S100A9 deletion.

### 3.8 Downregulation of S100A9 in the M/M cell line enhances efferocytosis by targeting the STAT6/PPARγ pathway

In the OGD/R HT22 and BV2 co‐culture system, S*100A9* silencing in BV2 cells significantly increased their engulfment of dead/dying cells (Pi^+^ HT22 debris), indicating enhanced clearance; however, the phenomenon was diminished by STAT6 phosphorylation inhibition (Supplemental Figure S4A,E). Pinocytosis is another phagocytosis essential for smaller molecule waste clearance in the brain. The FITC‐dextran uptake assay revealed that *S100A9* silencing enhanced the pinocytosis of FITC‐dextran by BV2. Additionally, inhibition of STAT6 phosphorylation hampered the uptake of FITC‐dextran in the absence of S100A9 (Figure S4B,F). Flow cytometry analysis also obtained similar results; PQD treatment increased the BV2 intracellular FIFC signal, indicating enhanced pinocytosis after S100A9 blockade, and was abrogated by AS treatment (Figure S5A–C).

Mechanically, the increased trend of phagocytosis and pinocytosis by *S100A9* knockdown or by blockage was attributed to S100A9 inhibition potentiated the phosphorylation of STAT6 and the downstream PPARγ (Figure S4D,G). As evidenced by the protein assay, the phagocytosis‐associated proteins, such as Arg1, TREM2, and CD36, increased while the M1 signature marker CD86 decreased accordingly after OGD/R (Figure S4H–K). Nevertheless, the expression of the phagocytosis‐associated protein was reversed by the treatment with the STAT6 phosphorylation inhibitor AS (Figure S4D,G–K), which confirmed the previous result that S100A9 inhibition was mediated by the STAT6/PPARγ signaling pathway.

### 3.9 Blocking the binding of S100A9 to TLR4 improved cerebral blood flow, reduced infarct area, and enhanced neurofunctional outcomes after tMCAO in wild‐type mice

**FIGURE 6:**
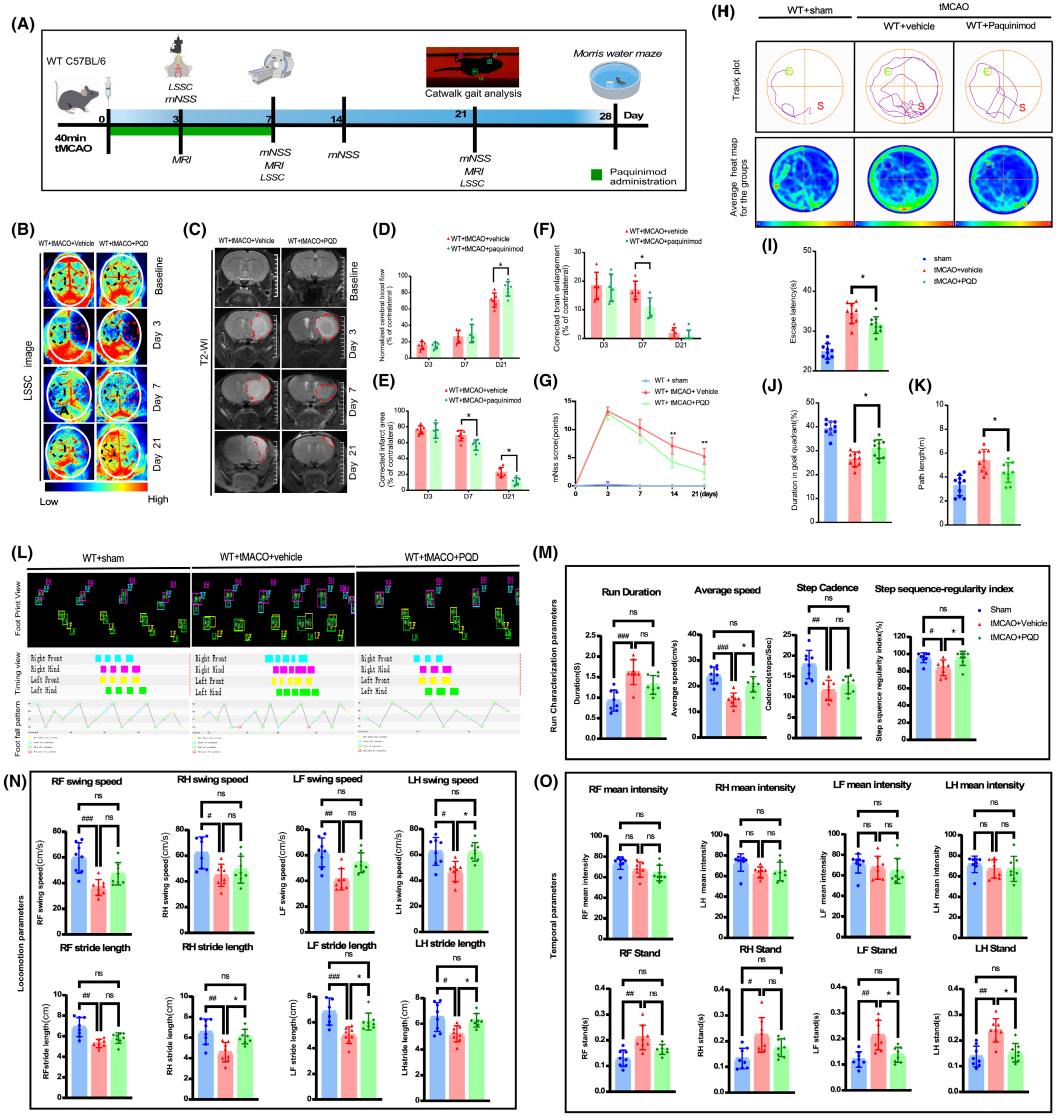
Treatment with PQD mitigates brain lesions and improves neurologic function after tMCAO. (A) A schematic diagram depicting the experimental procedure and observation time points. (B) Cerebral blood flow was assessed using laser speckle contrast imaging at baseline and at different time points in wildtype mice that received vehicle or PQD after tMCAO. Semi‐quantification was performed by normalizing the results to the same region of interest (ROI) in the contralateral hemisphere (D). *p < 0.05. (C) Representative MRI T2‐WI was used to assess the lesion area of the mice that received vehicle or PQD at baseline and at different time points after tMCAO. The corrected infarct volume (E) and brain enlargement (F) were normalized to the contralateral hemisphere. *p < 0.05. (G) Neurologic deficits were evaluated by the modified neurologic severity score (mNSS) of the treatment groups at different time points after tMCAO. **p < 0.01. (H) Representative track plot (top) and group track heatmap (bottom) of mice in each group in the Morris water maze test. Escape latency (I), duration in the target quadrant (J), and length of the swimming path (K) were quantified. *p < 0.05 vs. vehicle. (L) Representative prints, timing view, and footfall pattern of each group. (M) Run the characterization parameters in each group. Difference in average speed and step sequence regularity index. ^##^ *p* < 0.01 and ^###^ *p* < 0.001 vs. sham, *p < 0.05 *vs*. tMCAO+PQD. (N) Run the locomotion parameters in each group. The difference in left hind swing speed left hind and left front limb stride length. ^##^ *p* < 0.01 and ^###^ *p* < 0.001 *vs*. sham, *p < 0.05 *vs*. tMCAO+PQD. (O) Run temporal parameters in each group. Difference in standing time of the left front and left hind limb. ^##^ *p* < 0.01 and ^###^ *p* < 0.001 *vs*. sham, *p < 0.05 *vs*. tMCAO+PQD.

PQD is a compound that can modulate the immune system, and it is clinically valuable due to its safety, tolerability, and ability to prevent the binding of S100A9 to TLR4. WT tMCAO mice were treated with either PQD or vehicle for 7 consecutive days to determine their therapeutic potential in IS (Figure 6A). Although no significant differences in cerebral flow detected by LSC imaging exist between the two groups on days 3 and 7, however, when we extended the observation period, the PQD‐treated mice displayed a substantial decrease in neurologic deficits assessed by the mNSS score on days 14 and 21 (Figure 6G) and by cerebral flow measured by LSC imaging on day 21 (Figure 6B,D). PQD mitigated brain enlargement and reduced infarct volume, as detected by T2WI MRI on days 7 and 21, compared to the vehicle‐treated group (Figure 6C,E,F).

On day 21 after tMCAO, the catwalk gait analysis was performed to evaluate the different groups' motor sensation and coordination recovery (Figure 6L). Data show that PQD treatment significantly improved post‐stroke motor functions, as evidenced by a significant increase in the average run speed and the step‐sequence regularity index (Figure 6M), indicating mitigated regular step patterns and coordination movement. Furthermore, PQD treatment promoted functional recovery of the impaired limbs, as evidenced by increased left hindlimb swing speed and left‐sided foot stride length (Figure 6N). Although in the temporal parameters, there were no statistical differences in the mean intensity of the four‐limb footprints between the groups, notably, the PQD‐treated mice showed a shorter stand time of the left side limbs in each gait cycle than the vehicle group (Figure 6O). Moreover, the PQD treatment improved learning and spatial memory in the MWM test, as demonstrated by a shorter escape latency, path length, and extended goal quadrant duration time (Figure 6 I–K). Our preclinical study suggests that PQD treatment enhances cerebral blood flow, reduces infarct area, and promotes neurofunction and cognitive recovery in the tMCAO mouse model (Summarized in Graphical Abstract).

## 4 DISCUSSION

The present study first investigated the S100A9 expression pattern in the monocyte subpopulation of patients who have experienced acute IS. We provided the evidence for the first time that S100A9 is primarily expressed in the classical monocyte subpopulation, the S100A9 expression ratio was positively correlated with adverse long‐term clinical outcomes. Our results indicated that the S100A9 expression ratio in the classical monocyte maybe serve as a reliable biomarker for AIS prognosis predication. Additionally, we conducted experiments on newly generated S100A9 CKO mice to understand the role of S100A9 in M/M and its downstream signaling pathway in inflammatory response. We showed that the STAT6/PPARγ pathway was involved in S100A9 disruption, leading to M2‐like phenotype change and strengthening of efferocytosis. Interestingly, we discovered that PQD, a selective S100A9/TRL4 binding blocker, has been approved by the FDA for treating systemic scleroderma and has shown safety and efficiency in clinical trials treating other autoimmune/inflammatory diseases.^42^, ^48^ Here, we identified that PQD also effectively protected against neuronal damage and promoted functional recovery in an ischemic mouse model. Our research highlights the importance of S100A9 in regulating inflammation after IS and suggests that PQD could be a promising treatment option for patients with acute IS.

The role of inflammation in the aftermath of a stroke is complex. Depending on the stage of recovery, inflammation can have harmful or beneficial effects. When inflammation persists beyond its natural resolution, it can cause progressive brain damage and long‐term neurological deficits. Therefore, it is essential to have a therapeutic strategy in place to control the anti‐inflammatory and waste‐clearance properties of immune cells in the brain. This strategy is especially crucial for individuals who miss the “time window” of recanalization in the acute phase. If this strategy is successfully implemented, it could lead to promising outcomes in managing stroke‐induced complications.

Recent research indicates that high levels of plasma S100A9 are associated with various diseases such as diabetes, myocardial infarction, systemic lupus erythematosus,^49^ and ischemic stroke. One clinical study has shown that S100A9 represents a reliable biomarker in distinguishing acute progressive ischemic stroke from acute non‐progressive ischemic stroke,^50^ thus suggesting its potential role in ischemic pathophysiology. However, the expression of S100A9 in monocytes and its correlation with the prognosis of ischemic stroke is unknown. We showed that ‘classical’ monocytes primarily express intracellular S100A9 under normal conditions. However, after ischemic stroke, the proportion of ‘classical’ monocytes that were S100A9‐positive significantly increased and was associated with poor outcomes in patients with AIS.

In addition, S100A9 and its binding receptor TLR4 immunoactivity increased exclusively in Iba‐1^+^ M/M in the ischemic mouse brain. Therefore, S100A9 may harm ischemic stroke outcomes, and its function may be cell‐targeted. Previous studies suggest that a more abundant subset of classical monocytes (CD14^+^) triggers damaging effects, while less represented populations (CD16^+^) may exert beneficial functions.^51^ Hence, the downregulation of S100A9 may be helpful for neuroprotection. These findings support the potential of S100A9 as a biomarker for ischemic stroke and emphasize the importance of considering cell‐specific expression patterns when evaluating its therapeutic potential.

We performed in vivo experiments to investigate the role of S100A9 in the pathology of ischemic stroke. Deleting S100A9 in M/M had a potentially beneficial effect in mitigating brain damage and rescuing neurologic deficits. This protection could be caused by promoting anti‐inflammatory polarization of M/M and improving efferocytosis of dead or dying neurons, reducing white matter damage and further cell death.

Transcriptomics results revealed differential transcriptional profiles of phagocytosis and inflammatory cytokines in S100A9 null M/M mice isolated from ischemic brains compared to their wildtype littermates. It is important to note that STAT1, the STAT6 antagonist, was downregulated in the CKO tMCAO mouse brain. S100A9 downregulation significantly increased STAT6 phosphorylation, and the protective effects were diminished by the STAT6 phosphorylation‐specific inhibitor AS.

Our study outlined the expression time course of PPARγ in S100A9 CKO and wildtype mice after tMCAO for the first time. Although the PPARγ level peaked at approximately 3 days and decreased after that, S100A9 CKO significantly increased the PPARγ level compared to the control. Our in vivo study was reinforced by an in vitro experiment with the M/M cell line, which demonstrated that the STAT6 phosphorylation‐specific inhibitor AS reversed increased PPARγ. This result confirmed that PPARγ was a downstream signaling target of pSTAT6.

S100A9, a protein that plays a crucial role in inflammation modulation, has been the subject of research in the field. Despite this, the mechanisms underlying S100A9‐mediated M/M activation remain unclear. Signal transducers and transcription activators (STAT) are transcription factors that undergo phosphorylation by Jak kinase at a single tyrosine residue after interacting with the ligand‐receptor.^52^ Consequently, they assemble in a dimeric form, translocate to the nucleus, and bind to specific DNA sequence motifs.^46^ Recent studies have reported STAT1 and STAT6 as regulators of proinflammatory and anti‐inflammatory microglial phenotypes in cancer.^53^ STAT1 activation facilitates gene expression that encodes proinflammatory factors in *Cryptococcus neoformans*‐infected macrophages.^54^ Conversely, the loss of STAT1 in macrophages leads to an increased polarization of the M2 phenotype.^54^ In addition, TLR4 activation promotes macrophage redifferentiation by “turning on” polarization of the M1 phenotype while turning off polarization of the M2 phenotype, causing prolonged inflammation.^55^

Our study demonstrates that S100A9, the innate TLR4 ligand, facilitated STAT6 phosphorylation and PPARγ activation. The function of S100A9 was disabled by CKO in M/M in vivo, silenced by siRNA knockdown in vitro, or inhibited TLR4 binding by PQD treatment. Critical steps were identified by ablation of S100A9, and the processes were reversed by AS administration. Our findings confirm that STAT6 phosphorylation represents a crucial step in the ablation of S100A9.

Previous studies have documented that PPARγ plays a pivotal role in reducing inflammation, and treatment with PPARγ agonists may benefit intracerebral hemorrhage patients by reducing NF‐κB activation and cytokine levels.^47^ Additionally, preclinical studies have demonstrated that treatment with a PPARγ activator promotes the removal of red blood cells in vitro by stimulating CD36 expression,^56^ indicating its potential role in waste removal. In this regard, we have demonstrated that downregulation of S100A9 in M/M cell lines not only potentiates anti‐inflammatory factor expression but also promotes the activation of PPARγ, which in turn leads to an increase in the expression of the well‐characterized phagocytosis marker CD36.^57^ These results highlight the potential of PPARγ in promoting the phagocytic ability of M/M.

Our current research findings reveal that S100A9‐mediated M/M polarization exacerbates neuroinflammatory injury through the STAT6/PPARγ pathway in ischemic stroke. Furthermore, we have elucidated the molecular mechanism of potentiated efferocytosis by blocking or disrupting S100A9 binding to its receptor TLR4. Our results are consistent with previous reports that identified CD36 as a pivotal target of PPARγ‐mediated downstream gene transcription, whose expression is downregulated by TLR4 signaling in microglia and monocytes.^58^ Our findings suggest that PPARγ is an essential contributor to anti‐inflammatory responses, and treatment with PPARγ agonists may benefit ischemic stroke. Moreover, our research has identified that S100A9‐mediated M/M polarization aggravates neuroinflammatory injury via the STAT6/PPARγ pathway in ischemic stroke, providing a new understanding of the molecular mechanisms involved.

The present study has some limitations that require consideration. First, we tested the efficiency of PQD after IS using young, healthy mice as the stroke model. However, ischemic stroke typically affects older adults with hypertension or diabetes. Therefore, future studies should include different sexes and aged animals with comorbidities to strengthen the transnational potential. Second, we only focused on the function of S100A9 in M/M in ischemic stroke since these cells undergo specialized phagocytosis in the brain. However, we should address the function of neutrophils‐derived S100A9 in the perivascular area. However, neutrophils only survive briefly in the acute phase and cannot penetrate the glial network into the brain parenchyma after BBB leakage. A recent study has shown that neutrophil‐released S100A9 alters the platelet proteome in acute myocardial infarction (MI).^49^ Nonetheless, another study demonstrated that thrombus formation was reduced in whole blood from S100A9 global knockout mice, thus reducing the incidence of thrombotic diseases, including MI and stroke.^59^ Furthermore, a study developed a vaccine against S100A9 that inhibited thrombosis without increasing the risk of bleeding in ischemic stroke in mice.^26^ Our study elucidated that the neuroprotective effect of S100A9 disabling in ischemic stroke can be attributed to facilitating the shift of the M/M anti‐inflammatory phenotype and the efficiency of efferocytosis.

The current study confirms that downregulation of S100A9 in the M/M potentiates phagocytotic activity and promotes inflammation resolution in the ischemic brain. Additionally, it elucidated a mechanism whereby STAT6 and PPARγ work orchestrally to contribute to the poststroke protective responses mediated by S100A9 disruption. Notably, S100A9 blockade by PQD may be a promising therapeutic intervention in mitigating brain injury and promoting favorable outcomes after ischemic stroke.

## 5 CONCLUSIONS

The disruption of S100A9 in M/M ameliorates brain injury through the STAT6/PPARγ pathway in ischemic stroke. Targeting S100A9 through PQD may represent a novel and promising strategy for treating IS.

## AUTHOR CONTRIBUTIONS

JM and XL designed and conducted the main experiments. XL, JM, and JJ performed the MRI and imaging analysis. JM performed clinical study and data acquisition. XL, JM, and JW participated in the experiments and data analysis. XL, JM, JW, and JG participated in writing and revising the manuscript. All the authors have read, revised, and approved the final manuscript.

## CONFLICT OF INTEREST STATEMENT

The authors declare that they have no competing interests.

## CONSENT TO PARTICIPATE

Written informed consent was obtained from all participants before their inclusion in the study. For animal studies, all experiments were conducted according to the regulations of the Institutional Animal Care and Use Committee of Zhengzhou University.

## PATIENT CONSENT STATEMENT

All participants gave their written informed consent prior to their inclusion in the study.

## PERMISSION TO REPRODUCE MATERIAL FROM OTHER SOURCES

Not applicable.

## Supporting information


Figure S1.

Figure S2.

Figure S3.

Figure S4.

Figure S5.

Table S1.

Table S2.

Data S1.

## Data Availability

The datasets used and/or analyzed during the current study are available from the corresponding author upon reasonable request.
