# Supplementary figures and images for: S100A9 deletion in microglia/macrophages ameliorates brain injury through the STAT6/PPARγ pathway in ischemic stroke
Source: CNS Neurosci Ther. 2024 Aug 6;30(8):e14881. doi: 10.1111/cns.14881 (PMC11303267; doi:10.1111/cns.14881)

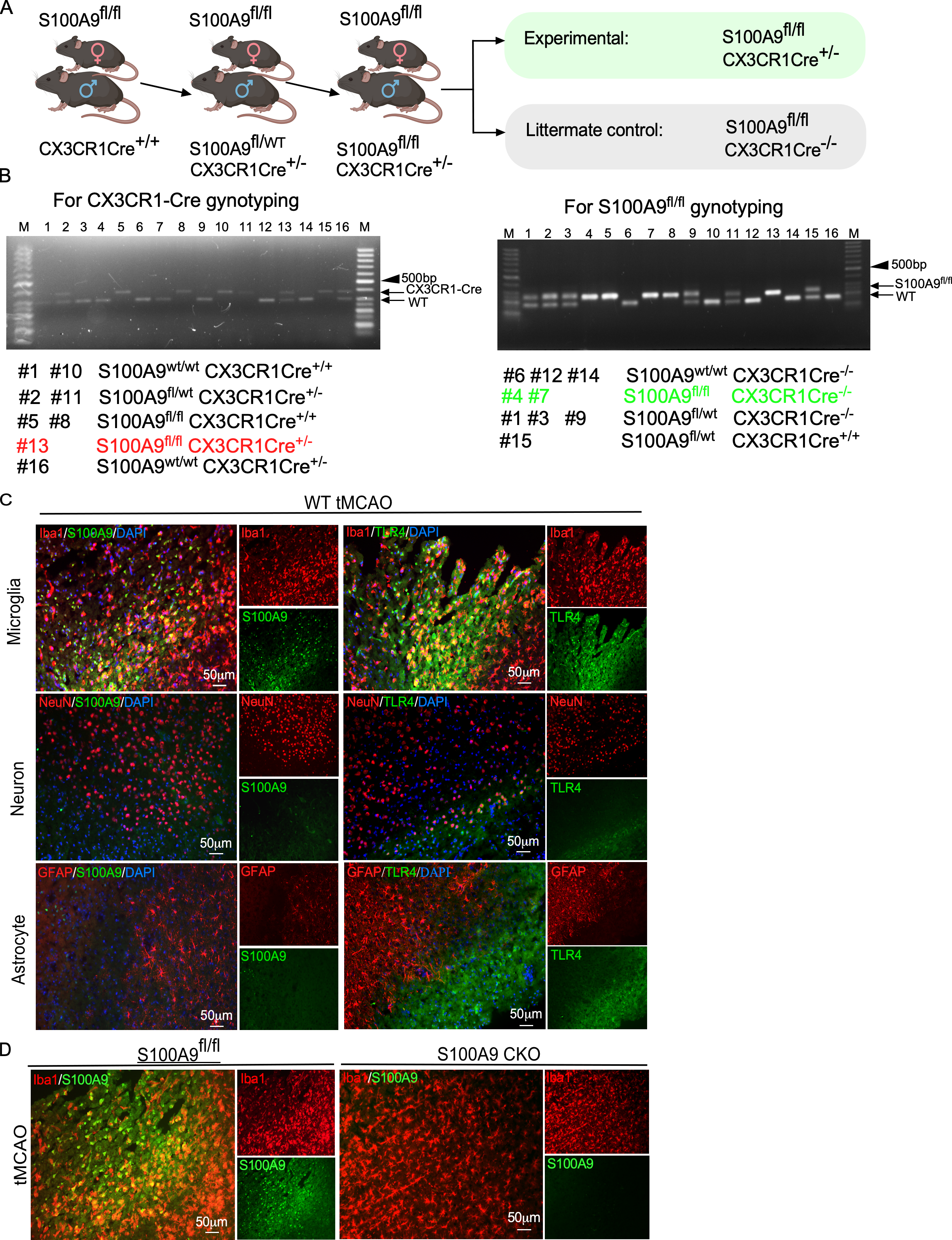

Supplement: Supplementary file 1 — Figure S1. [file CNS-30-e14881-s007.tif]

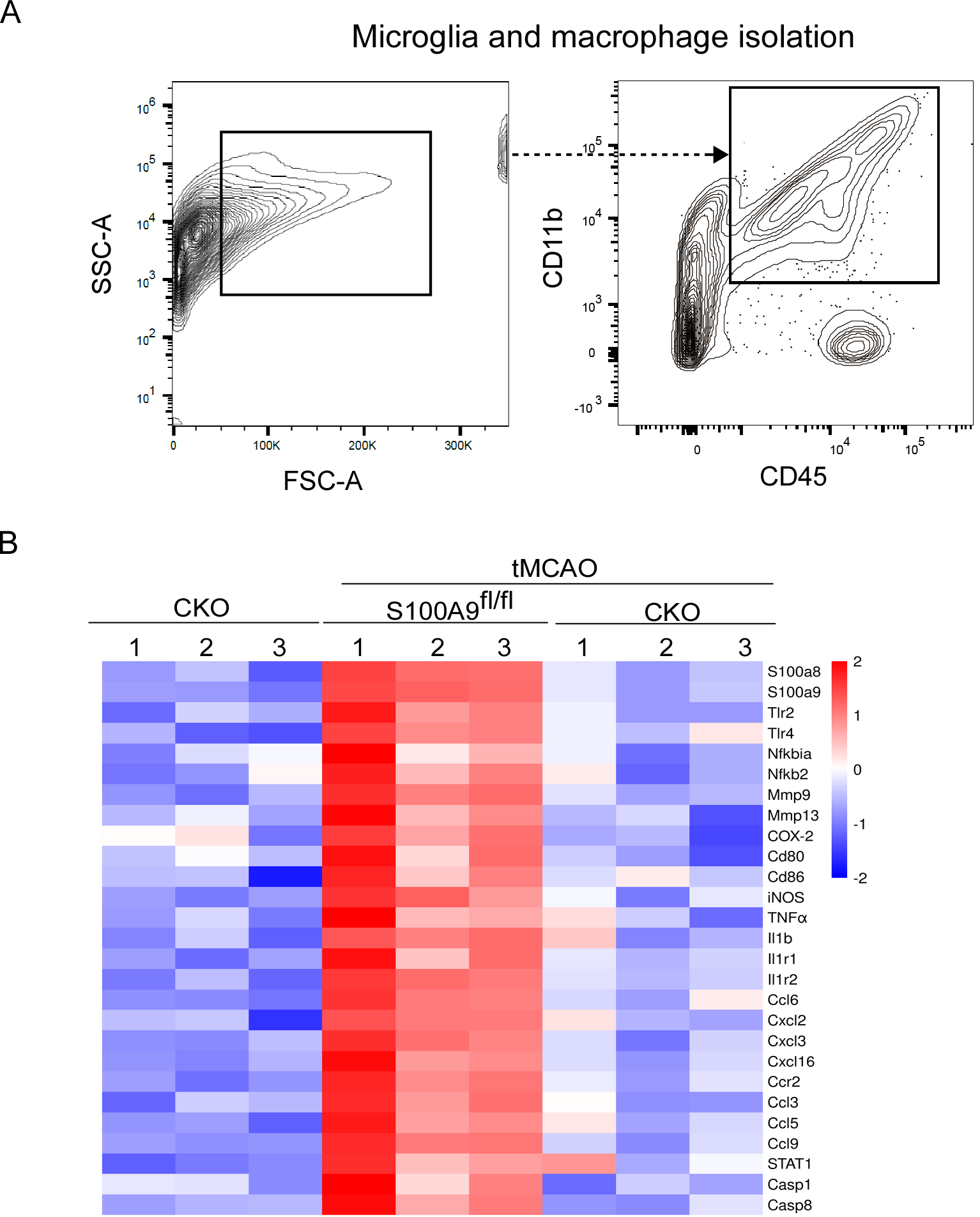

Supplement: Supplementary file 2 — Figure S2. [file CNS-30-e14881-s005.tif]

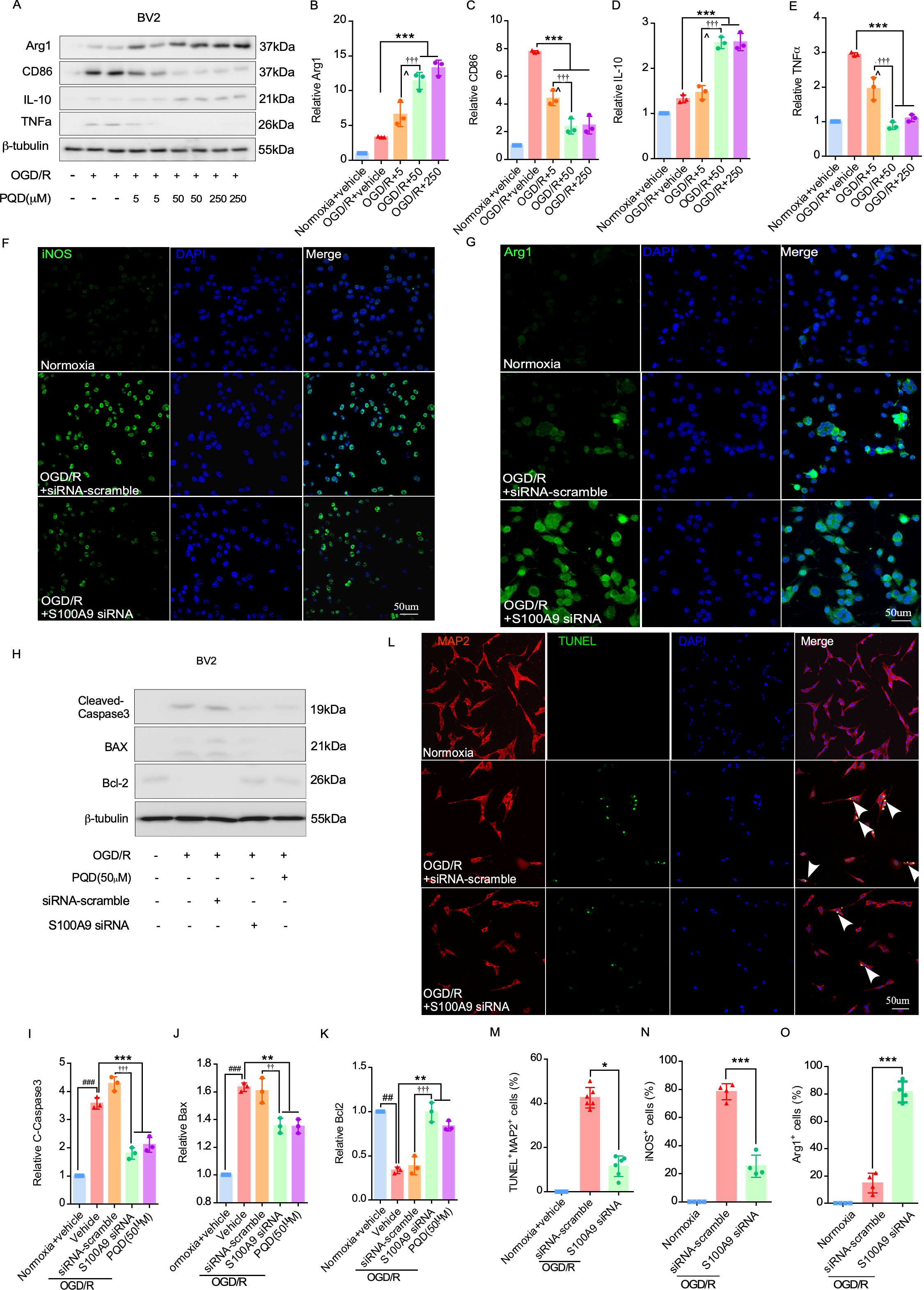

Supplement: Supplementary file 3 — Figure S3. [file CNS-30-e14881-s002.tif]

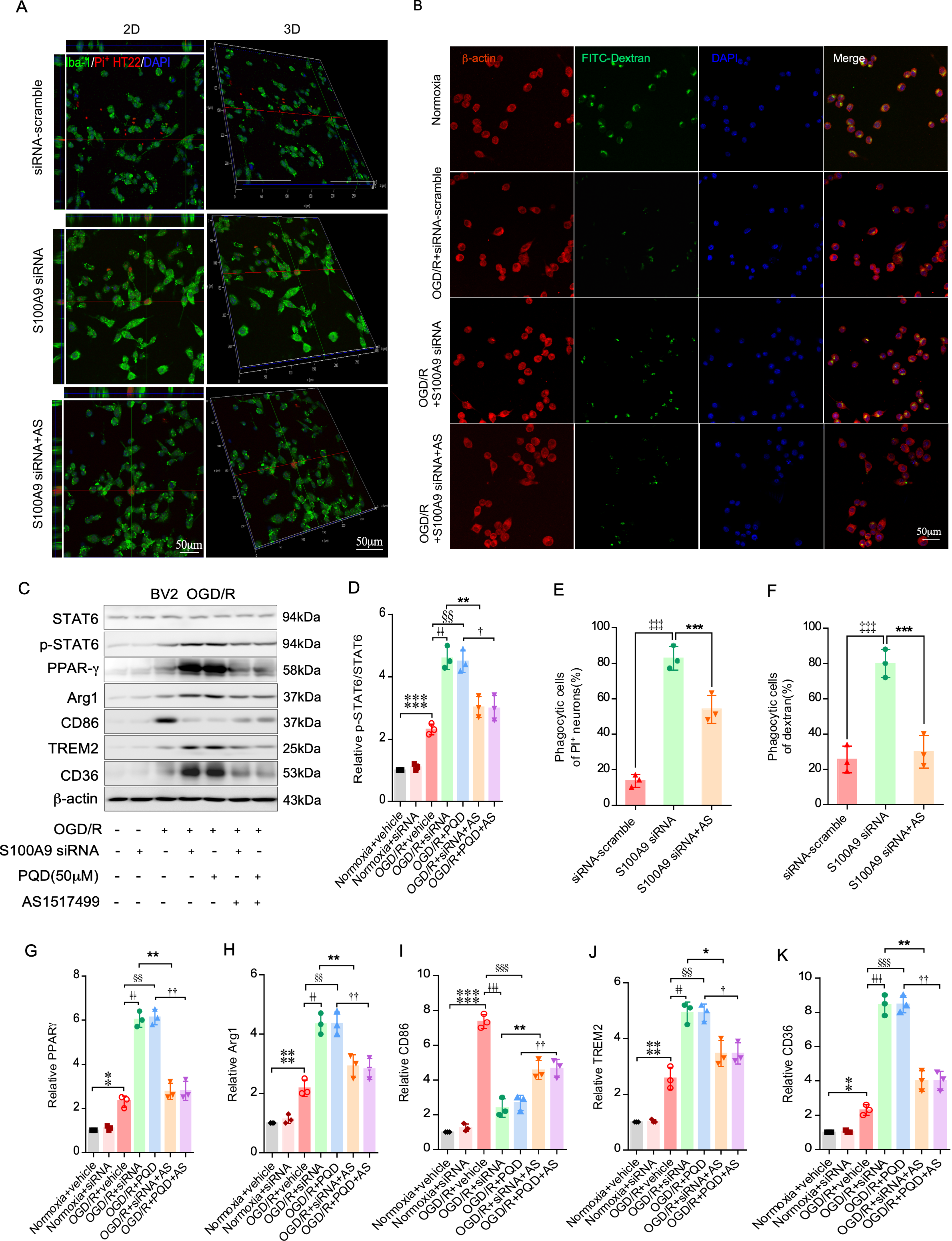

Supplement: Supplementary file 4 — Figure S4. [file CNS-30-e14881-s008.tif]

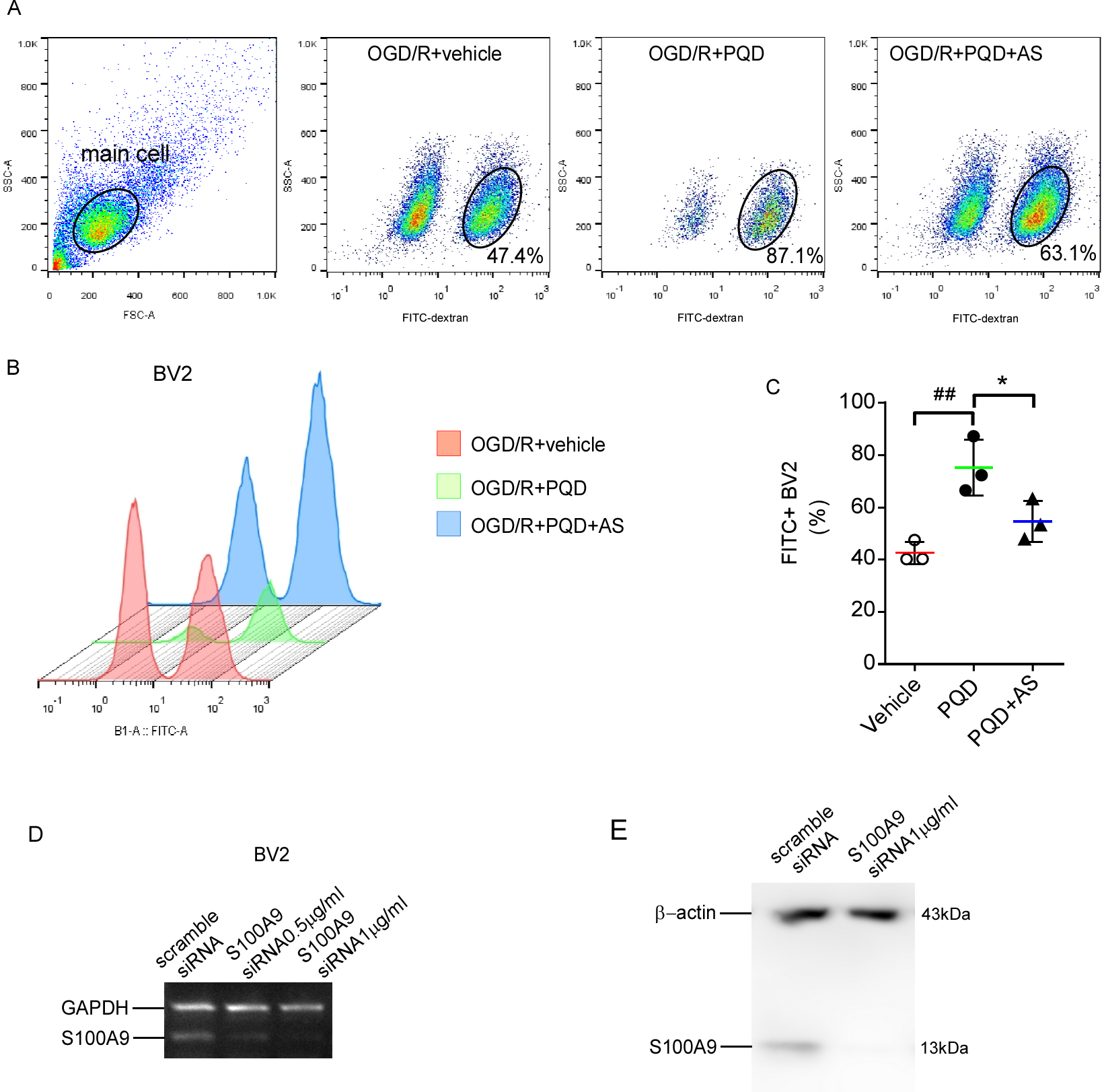

Supplement: Supplementary file 5 — Figure S5. [file CNS-30-e14881-s001.tif]
