# Supplementary material for: S100A9 deletion in microglia/macrophages ameliorates brain injury through the STAT6/PPARγ pathway in ischemic stroke
Source: CNS Neurosci Ther. 2024 Aug 6;30(8):e14881. doi: 10.1111/cns.14881 (PMC11303267; doi:10.1111/cns.14881)
Supplement: Supplementary file 6 — Table S1. [file CNS-30-e14881-s006.docx]

**Supplementary Table1. Details of the antibodies used in the experiment**

| Antibody | Brand | Catalog number | Host | Dilution |
| --- | --- | --- | --- | --- |
| NeuN | Abcam | ab177487 | Rabbit | 1:300 (IF) |
| GFAP | Abcam | ab7260 | Rabbit | 1:300 (IF) |
| MAP2  Iba1 | Sigma  Wako | M4403  011-27991 | Mouse  Goat | 1:300 (IF)  1:300 (IF) |
| MBP | Abcam | ab40390 | Rabbit | 1:500 (IF);  1:1000 (WB) |
| S100A9  TLR4 | Abcam  Santa Cruz | ab105472  sc-293072 | Rat  Mouse | 1:100 (IF)  1:200 (IF) |
| CD206 | Novus | NBP1-90020 | Rabbit | 1:300 (IF) |
| CD16/32 | Novus | NBP2-52644 | Rabbit | 1:300 (IF) |
| iNOS | Novus | NBP1-33780 | Rabbit | 1:300 (IF) |
| Arg1 | Novus | NBP1-32731 | Rabbit | 1:300 (IF);  1:1000 (WB) |
| TNFα | Novus | ab183218 | Rabbit | 1:1000 (WB) |
| IL10 | Affinity | DF6894 | Rabbit | 1:1000 (WB) |
| CD86 | Affinity | DF6332 | Rabbit | 1:1000 (WB) |
| STAT6 | Abcam | ab32520 | Rabbit | 1:1000 (WB) |
| STAT6 (phospho Y641)  PPARγ  Cleaved-caspase3 | Abcam  Cell signaling technology  Affinity | ab263947  #2435  AF7022 | Rabbit  Rabbit  Rabbit | 1:1000 (WB)  1:1000 (WB)  1:1000 (WB) |
| CD36 | Abcam | ab133625 | Rabbit | 1:1000 (WB) |
| TREM2 | Abcam | ab305103 | Rabbit | 1:1000 (WB) |
| BAX  Bcl-2 | Cell signaling technology  Abcam | #14796  ab182858 | Rabbit  Rabbit | 1:1000 (WB)  1:1000 (WB) |
| β-actin | Affinity | AF7018 | Rabbit | 1:500 (IF);  1:1000 (WB) |
| β-tubulin | Affinity | AF7011 | Rabbit | 1:1000 (WB) |
| GAPDH | Affinity | AF7021 | Rabbit | 1:1000 (WB) |
| Donkey Anti-Rabbit IgG H&L  Alexa Fluor[®](https://www.abcam.cn/donkey-rabbit-igg-hl-alexa-fluor-488-ab150073.html) 488  Donkey Anti-Rabbit IgG H&L  Alexa Fluor[®](https://www.abcam.cn/donkey-rabbit-igg-hl-alexa-fluor-488-ab150073.html) 594  Donkey Anti-Goat IgG H&L  Alexa Fluor[®](https://www.abcam.cn/donkey-rabbit-igg-hl-alexa-fluor-488-ab150073.html) 594  Donkey Anti-Mouse IgG H&L  Alexa Fluor[®](https://www.abcam.cn/donkey-rabbit-igg-hl-alexa-fluor-488-ab150073.html) 594  [Goat Anti-Rabbit IgG (H+L) HRP](https://www.affbiotech.cn/goods-6302-S0001-Goat_Anti_Rabbit_IgG_H_L_HRP.html)  FITC anti-human CD14  APC anti-human CD16  PE anti-human S100A9  APC anti-mouse CD45  FITC anti-mouse CD11b | Abcam  Abcam  Abcam  Abcam  Affinity  Biolegend  Biolegend  Biolegend  Biolegend  Biolegend | ab150073  ab150076  ab150132  ab150108  S0001  301803  302011  350705  103111  101205 | Donkey  Donkey  Donkey  Donkey  Goat  Mouse  Mouse  Mouse  Rat  Rat | 1:500 (IF)  1:500 (IF)  1:500 (IF)  1:500 (IF)  1:3000 (WB)  1:20 (FC)  1:20 (FC)  1:20 (FC)  1:400 (FC)  1:400 (FC) |
