# Supplementary material for: S100A9 deletion in microglia/macrophages ameliorates brain injury through the STAT6/PPARγ pathway in ischemic stroke
Source: CNS Neurosci Ther. 2024 Aug 6;30(8):e14881. doi: 10.1111/cns.14881 (PMC11303267; doi:10.1111/cns.14881)
Supplement: Supplementary file 7 — Table S2. [file CNS-30-e14881-s004.docx]

**Supplementary Table2. Details of the antibodies used in the experiment**

| Gene | PCR product length (Base pair) | Primer sequence |
| --- | --- | --- |
| *S100A9* | 114 | Forward :5′- CACAGTTGGCAACCTTTATGAA-3′ |
|  |  | Reverse :5′- TCATACACTCCTCAAAGCTCAG -3′ |
| *GAPDH* | 249 | Forward :5′-GCAGTGGCAAAGTGGAGATT -3′ |
|  |  | Reverse :5′-TCTCCATGGTGGTGAAGACA -3′ |
