# Supplementary material for: S100A9 deletion in microglia/macrophages ameliorates brain injury through the STAT6/PPARγ pathway in ischemic stroke
Source: CNS Neurosci Ther. 2024 Aug 6;30(8):e14881. doi: 10.1111/cns.14881 (PMC11303267; doi:10.1111/cns.14881)
Supplement: Supplementary file 8 — Data S1. [file CNS-30-e14881-s003.docx]

# **Supplementary Figure 1. Generation and genotyping/confirmation of S100A9 CKO mice.** (A) A schematic diagram illustrates the breeding procedure of S100A9 M/M condition knockout mice. The parental homozygous mouse CX3CR1Cre that carries the Cre enzyme coding sequence derived from the CX3CR1 promoter was matted with the maternal homozygous mouse that carries the two lox sequences on both sides of the S100A9 coding region. After the first round of genotyping screening, the heterozygous offspring F1 was matted with maternal homozygous F0 to generate the homozygous offspring F2 carrying homozygous S100A9lox and heterozygous CX3CR1Cre. Stable mouse colony was maintained by crossing S100A9^fl/fl^ CX3CR1 Cre^+/-^ with S100A9^fl/fl^ to generate S100A9 CKO mice (S100A9^fl/fl^ CX3Cr1 Cre^+/-^) or littermate control mice (S100A9^fl/fl^ CX3Cr1 Cre^-/-^). (B) The representative agarose DNA gel will show the genotyping result of the S100A9 lox or CX3CR1 Cre nucleotide fragments by PCR amplification. The homozygous S100A9lox, the heterozygous Cre-CX3CR1 (labeled in red), and the S100A9lox (labeled in green) were selected for use in the following experiments. (C) Immunolabelling of S100A9 and TLR4 and co-staining with the microglia marker Iba1 (upper panel), the neuronal marker NeuN (middle panel), and the astrocyte marker GFAP (lower panel) in WT mice 3 days after tMCAO. Arrows indicate that S100A9 and its TLR4 receptor were expressed exclusively in microglia, but not in the neuron or astrocyte, in response to tMCAO. (D) Confirmation of S100A9 knockout by co-staining the microglial marker Iba-1 and S100A9 in S100A9 CKO mice 3 days after tMCAO, S100A9^fl/fl^ mice served as a control. The image shows that the expression of S100A was completely abolished in the Iba-1^+^ cell after tMCAO (right panel).

**Supplementary Figure 2. Transcriptomic analysis revealed distinctive transcriptomic signatures of S100A9 CKO in tMCAO.** (A) Brain tissue in the peri-infarct areas of the S100A9^fl/fl^ and S100A9 CKO mice were harvested and subjected to FACS sorting. Gating stratagem to show the FACS cell sorting determined by the cell markers FITC-CD11b and APC-CD45 labeling. (B) Transcriptional analysis of the S100A9 null or wildtype M/M in the S100A9 CKO sham, S100A9 CKO tMCAO, and control tMCAO groups. Inflammation and apoptosis-associated markers were up-regulated in the control group compared to the CKO sham group and down-regulated in the CKO tMCAO group compared to the control group. The STAT1 gene associated with phagocytosis was negatively regulated in the S100A9 CKO group compared to the control group of tMCAO.

**Supplementary Figure 3. Downregulation of S100A9 potentiates the shift of the anti-inflammatory phenotype of BV2 cells and reduces neuronal death.** (A) The protein expression of M1-like phenotype markers, including Arg-1, CD86, Il-10, and TNF-a in BV2 cells exposed to OGD/R and treated with PQD at different dosages. (B-E) Quantification of Arg-1, CD86, Il-10, and TNF-a in BV2 cells with different treatments normalized to internal control. †††P<000.1 vs. OGD/R+5, ***P <0.001 vs. vehicle. Immunostaining of the M1-like marker iNOS (F) and the M2-like marker Arg1 (G) in BV2 cells after exposure to OGD/R and transfection with S100A9 siRNA (lower panel) or scrambled siRNA (middle panel). The removal of S100A9 by siRNA also decreased the expression of iNOS (N) and increased the expression of Arg1 (O). ‡‡‡*P*<0.001 vs. scrambled siRNA. (H) Western blot gels show the protein expression of apoptotic proteins, such as cleaved caspase-3, BAX, and BCL2, in BV2 cells exposed with/without OGD/R and subjected to various treatments. Quantification of normalized relative cleaved caspase-3 (I), BAX (J) and Bcl-2 (K). Treatment with S100A9 and siRNA PQD increased cleaved caspase3 and BAX expression but decreased Bcl2 expression. ###*P*<0.01*vs.* normoxia+vehicle. ***P*<0.01, ****P*<0.001 vs. vehicle. ‡‡*P*<0.01, ‡‡‡*P*<0.001*vs.* scrambled siRNA. (L) Co-immunostaining of MAP2 and TUNEL in HT22 cells co-cultured with BV2 cells transfected with scrambled siRNA (middle panel) or siRNA S100A9 (lower panel) and exposed to OGD/R. (M) Quantification of the rate of neuronal death by MAP2+TUNEL+/total MAP2+. ‡*P*<0.05 *vs.* scrambled siRNA.

**Supplementary Figure 4. Downregulation of S100A9 enhances the phagocytotic function of BV2 cells in a STAT6/PPARγ-dependent manner.** (A) In the phagocytosis assay, Iba-1-labeled BV2 cells transfected with scrambled siRNA (top) or S100A9 siRNA (middle) with vehicle or STAT6 phosphorylation inhibitor AS were subjected to Pi-labelled apoptotic HT22 cells. A 3D image (right lane) was used to confirm that Pi^+^ cells were taken inside BV2 cells. The phagocytosis assay was quantified in (E). (B) Pinocytosis assay of Iba-1-labeled BV2 cells subjected to OGD/R and exposed to FITC-conjugated dextran 40,000 and quantified in (F), *^***^P*<0.001 *vs.* vehicle, ^‡‡‡^*P*<0.001 *vs.* scrambled siRNA. (C) Western blot gels show the protein expression of STAT6, p-STAT6, Arg1, CD86, TREM2 and CD36 in BV2 cells transfected with S100A9 siRNA and subjected to various treatments. S100A9 silencing or PQD treatment in BV2 cells significantly increased p-STAT6 (D) and phagocytosis-associated proteins such as PPARγ (G), Arg1 (H), CD36 (K), and TREM2 (J), but decreased the expression of the M1 signature marker CD86 (I). The expression pattern was reversed by AS treatment. ^*^*P*<0.05, ^**^*P*<0.01, ^***^*P*<0.001 *vs*. vehicle ，^⁑^*P*<0.05, ^⁑⁑^*P*<0.01, ^⁑⁑⁑^*P*<0.001 *vs*. normoxia + vehicle, ^§^*P*<0.01, ^§§^*P*<0.001 *vs*. PQD, †*P*<0.05, **^††^***P*<0.01 *vs.* PQD+AS. ^ǂǂ^ *P*<0.01 ^ǂǂǂ^*P*<0.001 *vs.* S100A9 siRNA.

**Supplementary Figure 5. S100A9 blockage increased pinocytotic function of BV2 pinocytotic function and was reversed by inhibition of STAT6 phosphorylation.** Flowcytometry analysis of the intracellular FITC signal in BV2 (A) subjected to OGD/R and treated with PQD or PQD + AS and exposed to FITC-Dextran 40,000. A representative histogram image shows the portion of the FITC^+^ population in BV2 (B) , depending on the intensity of the intracellular FITC signal. Quantifying the relationship of the FITC^+^ population cell count with the total BV2 (C) cell count in different treatment groups. ^##^*P*<0.01, ^###^ *P*<0.001, vs. vehicle, **P*<0.05, ***P*<0.01 vs. PQD+AS. (D) RT‒PCR, Western blotting (E) confirmed the successful silencing of S100A9 by siRNA knockdown. GAPDH or β-actin was used as an internal control.
